# Selenonium Ylides: Syntheses, Structural Aspects, and Synthetic Applications

**DOI:** 10.3390/molecules25102420

**Published:** 2020-05-22

**Authors:** Józef Drabowicz, Aneta Rzewnicka, Remigiusz Żurawiński

**Affiliations:** 1 Division of Organic Chemistry, Centre of Molecular and Macromolecular Studies, Polish Academy of Sciences, Sienkiewicza 112, 90-363 Łódź, Poland; 2 Institute of Chemistry, Jan Dlugosz University in Czestochowa, Armii Krajowej 13/15, 42–200 Czestochowa, Poland

**Keywords:** selenonium ylides, selenonium salts, chirality, stereogenic selenium atom, asymmetric synthesis, optical resolution, reactivity

## Abstract

The goals of this mini review constitute (a) a presentation of the synthetic protocols applied to the preparation of achiral and non-racemic selenonium ylides; (b) discussion of their basic structural features, including their optical activity; and (c) a description of their synthetic applications in general synthetic methodology and in asymmetric synthesis.

## 1. Introduction

**Figure 1:**
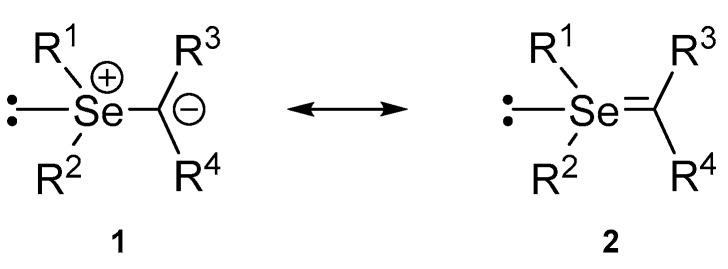
Resonance structures of selenonium ylides **1** and **2**.

A large family of organic selenium derivatives showing various reactivities and structural properties can be classified by different criteria. A general systematic scheme proposed by Martin and coworkers [1,2], based on simultaneous consideration of the formal oxidation state and number (*N*) of ligands bonded to selenium, allows a description of selenonium ylides, for which two resonance structures **1** and **2** can be considered, as 8-Se-3 or 10-Se-3 species, respectively (Figure 1).

It is worth noting that for this family of selenoorganic derivatives, adopting resonance structure **1** allows formal consideration of these compounds as the group of trivalent tri-coordinated onium salts. On the other hand, considering structure **2**, in which the central selenium atom has expanded its valence shell from 8 to 10 electrons, allows them to be treated formally as hypervalent compounds (concept first proposed by Musher in 1969 [3]). Due to the importance of the charged structure **1,** polarized from selenium to carbon, selenonium ylides are not considered as hypervalent compounds and they represent 8-Se-3 species. In our review, both resonance structures of selenonium ylides are used.

**Figure 2:**
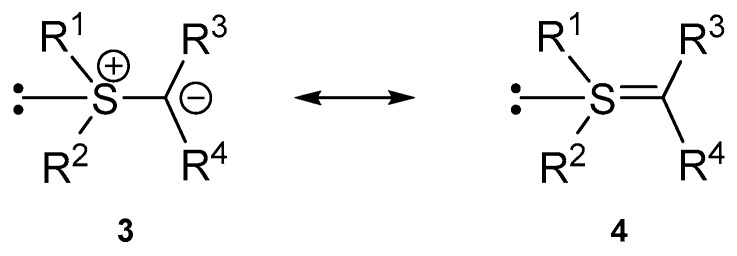
Resonance structures of sulfur ylides **3** and **4**.

After looking at the structure of sulfonium ylides (Figure 2), which constitute the basic group of sulfur ylides [4,5,6,7,8], it is obvious to consider them as a reference point for selenonium ylides (Figure 1). This is due to the fact that their reactivity is dominated mainly by the presence of a highly polarized heteroatom–carbon bond, and their chirality is associated with their tetrahedral geometry, which induces the optical activity of compounds in which three different carbon chains or/and rings are bonded to a stereogenic heteroatom.

**Figure 3:**
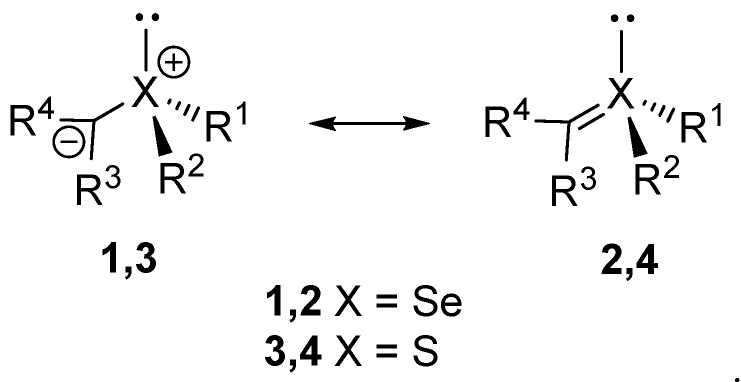
Stereo presentation of resonance structures of selenium(sulfur) ylides **1**–**4**.

The tetrahedral geometry of chiral sulfur and selenium ylides can also be represented alternatively as an irregular trigonal pyramid in which the selenium atom occupies the top of the pyramid (Figure 3).

In fact, studies devoted to sulfur ylides, especially optically active derivatives, are much richer compared to selenium analogues [4,5,6,7,8]. The best illustration of this phenomenon may be the comparison of the number of documents, which can be obtained by entering the terms “sulfur ylides” and “selenium ylides” or their general drawings to SciFinder or Reaxys databases (close 1 to 6, i.e., around 500 for sulfur ylides and 85 for selenium ylides).

The purpose of this mini review is to provide, as much as possible, available information on the preparation of selenium ylides, including the isolation of optically active species, their chemical and optical stabilities, basic reactivity, and their use in organic and asymmetric synthesis. It is our hope that this paper will stimulate additional research on this topic. It should be noted here that this topic has not yet been presented in a dedicated review. It has usually been discussed as a part of review articles or book chapters devoted to the chemistry of organic chalcogen derivatives, the co-authors of which were researchers conducting research in this field. A list of such, usually short, literature compilations opens a mini review based on the lecture given by Lloyd and published in a special issue of Chemica Scripta devoted to selenium chemistry in 1975 [9]. For the next few years in a row, a brief discussion devoted to selenonium ylides was included into the chapters from Specialist Periodical Reports, Organic Compounds of Sulfur, Selenium, and Tellurium [10,11,12,13,14]. A few years later, similar discussion was included into Chapter 17 of The Chemistry of Organic Selenium and Tellurium Compounds from Patai’s Chemistry of Functional Groups [15]. An update of this information can be found in an analogous chapter entitled “Synthesis of selenium and tellurium ylides and carbanions: application to organic synthesis” again included in “The Chemistry of Organic Selenium and Tellurium Compounds” from Patai’s “Chemistry of Functional Groups” series published in 2014 [16]. There are also brief accounts that describe experiments on the synthesis, stereochemical aspects, and the application in asymmetric synthesis of optically active tricoordinated selenium and tellurium compounds in which optically active selenonium ylides are also mentioned [17,18]. A piece of information related to the chemistry of selenonium ylides can be found in a review article entitled “Twice stabilized chalcogen ylides” written in the Russian language and published in Uspekhi Khimii in 1981 [19]. Below, we are going to discuss the synthesis of selenonium ylides (achiral, racemic, and optically active), their structural parameters and selected reactivities, and their application in general and asymmetric syntheses.

## 2. Structural Features

**Scheme 1:**
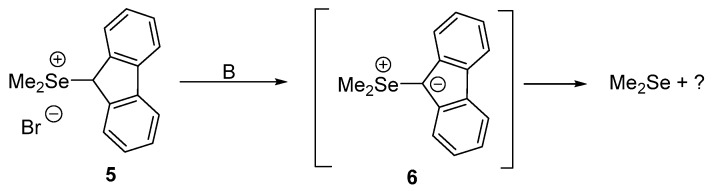
Unsuccessful attempt to isolate fluorenyl-9-dimethyl selenonium ylide **6**.

The basic geometrical parameters of selenonium ylides (tetrahedral arrangement of three substituents and a lone electron pair) were not mentioned in two pioneering papers devoted to this group of selenoorganic derivatives. In fact, the first attempt to isolate selenonium ylide as a stable chemical entity was unsuccessful. It was found that the action of aqueous alkali or ammonia on fluorenyl-9-dimethylselenonium bromide **5** gave the expected selenonium ylide **6** as a black solid product, which after dissolving in organic solvents, decomposed rapidly with the evolution of dimethyl selenide even at room temperature (Scheme 1) [20].

**Figure 4:**
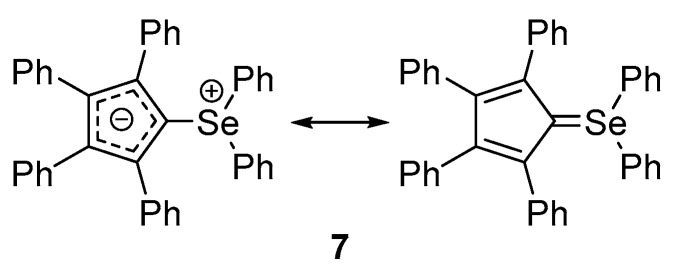
The first isolated selenonium ylide **7**.

It was not until more than 30 years ago that the first stable selenonium ylide **7** (Figure 4) was isolated and fully characterized (for details, see Section 3—Synthesis) [21]. However, also in this article, the geometrical features of a chemically stable compound are not mentioned.

**Figure 5:**
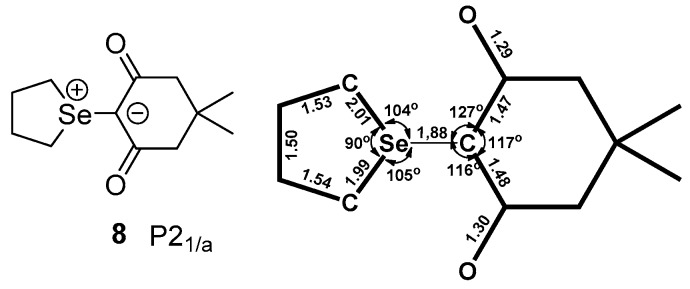
Selected cystallographic data of selenonium ylide **8** (taken from [22]).

Only the first crystallographic data obtained in 1972 for the selenonium ylide **8** fully supported the aforementioned geometrical framework of this family of ylides (Figure 5) [22].

**Figure 6:**
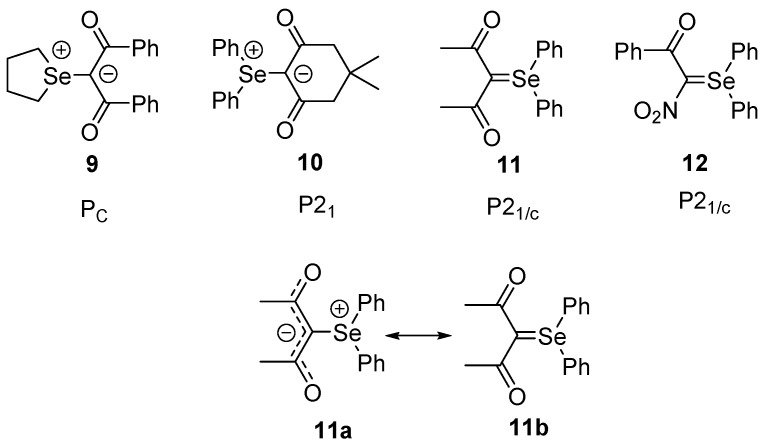
Selenonium ylides **9**–**12**.

Namely, it was found [22] that in the structure of this solid ylide, which crystallizes in the P2_1_/a space group, the selenium atom occupies the top of the irregular trigonal pyramid with angles C-Se-C 90, 104, 105°. The value of the first angle (90°) results from the rigidity of the cycle, as in the free ion (CH_3_)_3_Se^+^ the corresponding angles are 98, 98, and 99° [23]. The distance Se-C (endocyclic) 1.88 Å practically does not differ from the length of the corresponding bonds in dibenzoselenophene 1.90 Å [24]. It is shorter than the weighted average distances Se-C_arom_ 1.92(2) Å and Se-C_alif_ 1.98(2) Å [24], as well as distances Se-C 1.95 Å in trimethylselenonium iodide [23] but longer than a double Se=C bond of 1.80 Å in the structure of [1,2,5]oxaselenazolo[2,3-*b*][1,2,5]oxaselenazole-7-Se^IV^ [25]. Over the next few years, the determination of the molecular geometry was achieved by an X-ray structure analysis for two other cyclic selenonium ylides **9** and **10** [26] and acyclic diacetylmethylenediphenylselenurane **11** [27] and acetylnitromethylenediphenylselenurane **12** [28] (Figure 6). Similar parameters were observed in all cases.

Thus, the detailed analysis [27] of the data collected for the orange crystals of **11** showed that they are monocyclic with four molecules in the unit cell. The space group is P2_1_/c and the configuration at selenium in **11** is pyramidal with Se lying 0.78 Å out of the plane of the three substituents. The Se-C[C(CO)Me)]_2_ bond length is 1.906(8) Å, and the Se-C(phenyl) bond lengths are 1.898(9) and 1.926(9) Å. The long Se-C[C(CO)Me)]_2_ bond and the configuration at selenium was suggested to indicate a large contribution of the ylide resonance structure **11a**. The importance of resonance structure **11a**, in which the delocalization afforded the carbanion by the carbonyl groups, is evident from the IR spectrum of the selenonium ylide **11**, in which the carbonyl group gave a broad absorption at 1520 cm^−1^.

**Figure 7:**
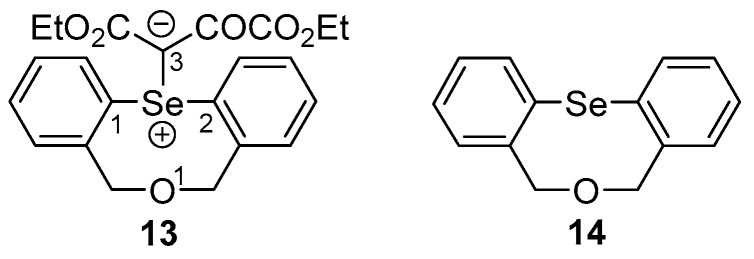
Selenonium ylide **13** and its precursor **14**.

The X-ray structural analysis of a remarkably stable crystals of the selenonium ylide **13** (monoclinic, space group P2_1_) (Figure 7) [29] shows the strong transannular contact between oxygen and selenium atoms [O(1)-Se contact is 2.70 Å]. The approximately collinear C(3)-Se-O(1) angle of 170.3° indicates that the cationic species generated on the selenium atom strongly interacts with the oxygen atom of the ether linkage. In addition, the bond length of Se-C(3), 1.887 Å, supports a strongly polarized resonance structure (since the calculated double Se-C bond is close to 1.84 Å [30,31] and the C(1)-Se-C(2) angle, 107.7°, supports the pyramidal geometry.

**Figure 8:**
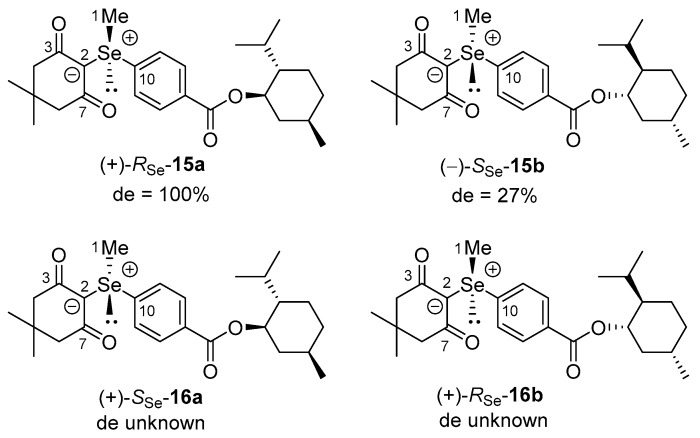
Diasteroisomerically pure selenonium ylide **15a** and diastereoisomerically enriched ylides **15b** and **16a,b**.

From a set of four diastereomeric selenonium ylides, [(menthyloxycarbonyl)phenyl] (methyl)selenonium 4,4-dimethyl-2,6-dioxocyclohexylides **15a,b** and **16a,b** prepared with the use of (−)-(1*R,*2*S,*5*R*)-menthol or (+)-(1*S,*2*R,*5*S*)-menthol as a chiral auxiliary (Figure 8) diastereoisomerically pure, dextrorotatory selenonium ylide, (+)-{4′-[(−)-menthyloxycarbonyl]}phenyl](methyl)selenonium 4,4-dimethyl-2,6-dioxocyclohexylide **15a,** was isolated by multiple fractional recrystallizations [27,28]. The X-ray crystallographic analysis allowed determination of its *R* absolute configuration at the stereogenic selenium atom. [32,33]. This analysis also showed that the bond length Se-C(2) (1.873 Å) in this diastereoisomer is much shorter than the standard Se-C bond length (1.95–2.04 Å) [25,26] and slightly shorter than that observed in diphenylselenonium diacetylmethylide **11a** (1.906 Å) [27]. This suggests the important resonance contribution from an ylene structure in ylide **l5a**. This is additionally supported by the shortening of the bond lengths C(2)-C(3) and C(2)-C(7) (1.41 Å) in isomer **l5a**. They are shorter than the usual C-C single-bond [25,26]. The bond angles around the stereogenic selenium in **l5a** equal to C(1)-Se-C(2) 103.8(4)°, C(1)-Se-C(l0) 98.6(4)°, and C(2)-Se-C(10) 102.1(4)° are smaller than the values of 100.8, 105.0, and 107.5° observed in diphenylselenonium diacetylmethylide **11** [27]. These changes suggest that selenonium ylide **l5a** has a ‘sharper’ pyramidal structure than diacetylmethylide **11** [27].

**Figure 9:**
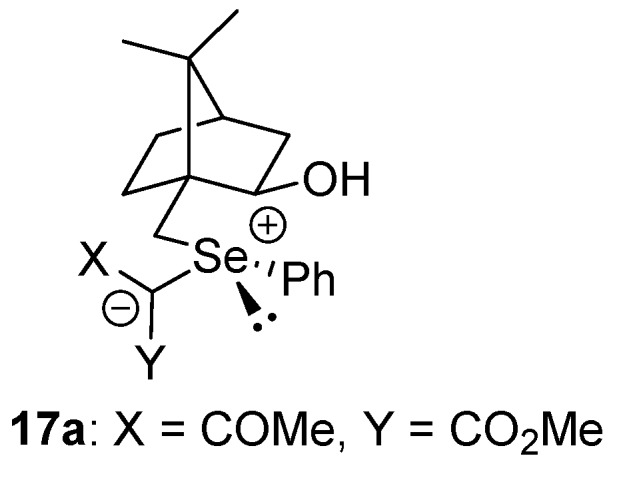
Selenonium ylide **17a** containing the 2-*exo*-hydroxy 10-bornyl moiety.

The X-ray crystallographic analysis also allowed determination of its *R* absolute configuration at the stereogenic selenium atom in the selenonium ylide **17a** designed using the 2-*exo*-hydroxy 10-bornyl moiety as a chiral frame (Figure 9) [34]. This analysis also showed that the bond length and bond angels of **17a** are similar to those presented above for **15a**.

**Figure 10:**
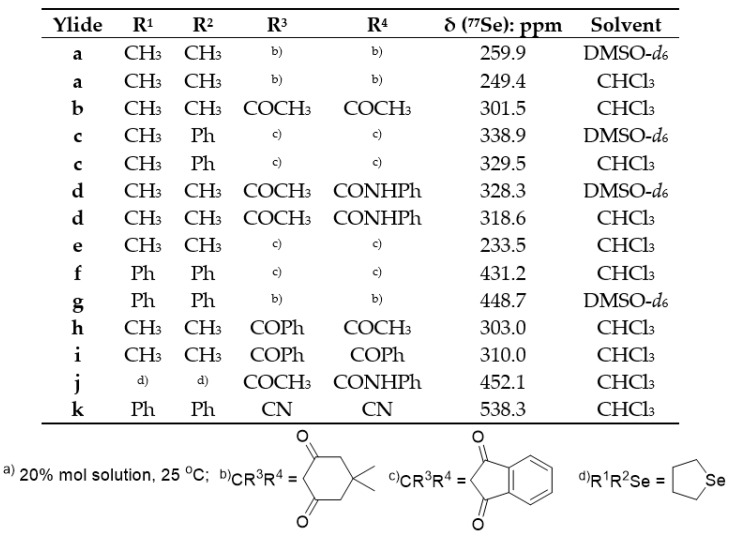
^77^Se chemical shifts of selenonium ylides **1**^a)^ (R^1^R^2^Se^+^-^-^CR^3^R^4^) (Me_2_Se as a reference).

**Figure 11:**
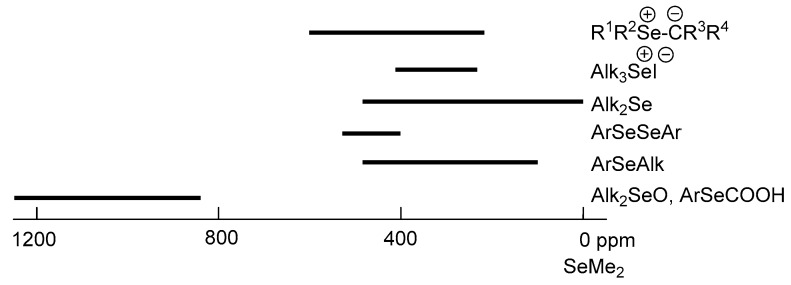
Figure presenting the relationship between the structure and chemical shift for the different groups of selenoorganic derivatives.

In addition to the use of X-ray analysis to confirm the solid state structures of selected selenonium ylides, including the assignment of the absolute configuration of a stereogenic selenium atom of two optically active diastereoisomerically pure ylides, other spectroscopic techniques were used and are still applied in the determination of other structural features of achiral and chiral selenonium ylides. Of the various spectroscopies used for these purposes, ^77^Se-NMR spectroscopy can be formally considered as natural. Its utility, however, is limited because it only allows the incorporation of the tested compound into a given family of selenoorganic derivatives, taking into account its chemical shift. This is most probably the reason why there is only one publication, in the available chemical literature, devoted specifically to ^77^Se-NMR spectroscopy. The basic topic of this paper, published in Russian as early as 1982, is a brief discussion on the relationship between the structure of selected selenonium ylides and their ^77^Se chemical shift [35]. The table prepared based on the original data is placed below (Figure 10). We also prepared a plot showing the relationship between the chemical shift and the structure of various selenoorganic groups (Figure 11).

The analysis of the relationship between the structure and chemical shift for the various groups of selenoorganic derivatives shown in both figures indicates that the range of ^77^Se chemical shifts observed for selenonium ylides is not very characteristic for this group of selenoorganic derivatives, because it is imposed with the range that is characteristic for trialkylselenonium iodides, dialkyl selenides, alkyl aryl selenides, diaryl diselenides, and aryl alkyl selenides. In this context, it is interesting to note that the ^77^Se chemical shift for the selenonium ylide **13** in CDCl_3_ shows a downfield shift to σ = 504.1 from σ = 367.1 observed for its precursor **14** (relative to Me_2_Se) [24].

As a rule, ^1^H-NMR spectroscopy is used as a standard tool for the structural determination of selenonium ylides, which provides information on the numbers of chemically nonequivalent protons, which appear in the spectra of given compounds as typical multiplets. However, sometimes, in addition to standard applications, it is also used to solve more specific structural problems associated with, for example, dynamic processes occurring in the test molecule or to determine the enantiomeric or diastereomeric excesses of chiral derivatives. Thus, based on the ^1^H-NMR spectrum of the selenonium ylide **13,** in which the methylene protons peaks absorb at σ = 4.79 and 5.03 (ABq *J* = 14.0 Hz), the existence of the boat conformation of an eight-membered selenium-containing ring was suggested [29].

**Figure 12:**
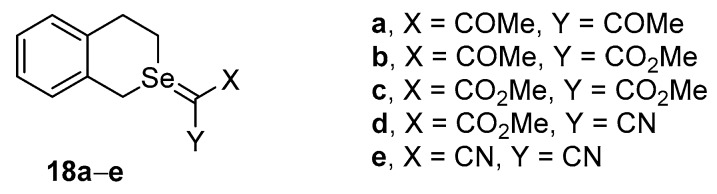
Cyclic *exo*-selenonium ylides **18a**–**e** based on the dihydro-2-benzoselenin cycle.

The lack of broadening of the methyl peak in the ^1^H-NMR spectrum of the selenonium ylide **11** upon cooling to −60 °C indicates that the equilibration of the two methyl groups proceeds via an energy barrier of <10 kcal/mol [27]. This value is lower than that of the sulfonium ylide Me_2_SC(COCMe)_2_ for which a barrier of 12 kcal/mol (based on C-methyl signals broadening at −25 °C) was reported [36]. The ^1^H-NMR spectra were used for the determination of the diastereoisomeric excesses of selenonium ylides **15a** and **15b**. Unexpectedly, only one singlet signal of MeSe group was observed, at δ **=** 3.22, not only in diastereoisomerically pure ylide (+)-**15a** but also in diastereoisomeric mixture of selenonium ylides **15a**,**b**. However, two singlets for the MeSe group were observed, at δ **=** 4.01 and 4.16, for the diastereoisomeric mixture of selenonium ylides **15a**,**b** when the ^1^H-NMR spectrum was recorded in the presence of europium trisheptafluorobutyrylcamphorate [Eu(hfc)_3_] as a chiral shift reagent. Moreover, the *ortho*-aromatic hydrogen (from Ar-selenium ring) in diastereoisomers **15a**,**b** appeared as two AB quartets at δ = 7.724 and 7.732. Thus, the diastereoisomeric purity of the diastereoisomeric mixture of ylide **15** was determined from the integration of their ^1^H-NMR signals at the ortho-position of the aromatic ring or at the MeSe signal using the shift reagent [32]. The analysis of the ^1^H-NMR spectra of the cyclic *exo*-ylides, (3,4-dihydro-1*H*-2-benzoselenin-2-io)methanides **18a**–**e** (Figure 12), which showed AB quartets assigned to 1-H of the methanide entities occupying the sterically relaxed pseudo-equatorial positions, allowed the suggestion of the preference for the given conformation of the 6-membered saturated selenocyclohexane, as the protons *cis* to the 1- and 4-positions of the methanide groups appear at lower magnetic fields as a result of the anisotropic effects of the methanide entities [37].

**Figure 13:**
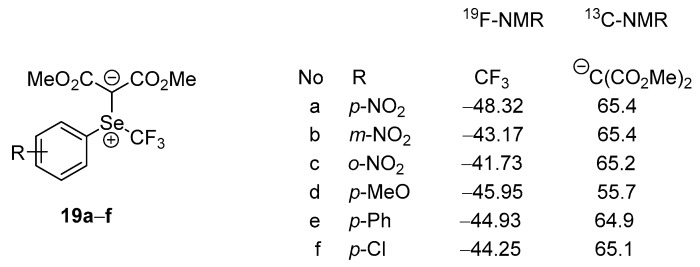
**r**^13^C ^r^and ^19^F chemical shift of selenonium ylides **19a**–**f**.

There are only very few reports on the application of ^13^C- and ^19^F-NMR spectroscopies as a standard tool for the structural determination of selenonium ylides, which provides information on the numbers of chemically nonequivalent carbon or fluorine atoms, which appear in the spectra of given compounds as typical multiplets. The data for a series of selenonium ylides **19a**–**f**, which were extracted from the supporting information of a very recent publication [38], are given in Figure 13. Similar values were observed for the ethyl analogue of the selenonium ylide **19a** [39].

**Figure 14:**
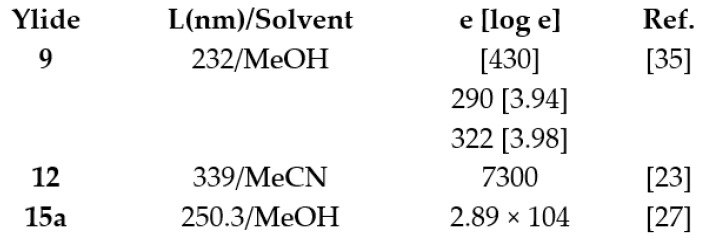
UV data for selenonium ylides, **9**, **12**, and **15a**.

The application of IR and UV-VIS spectroscopies also has a standard character for the structural determination of selenonium ylides, which provides information on the incorporation of basic functional groups (carbonyl and aromatic rings) in a given structure. Thus, the carbonyl groups of selenonium ylide **11** gave a broad IR absorption at 1520 cm^−1^. This value is strongly shifted to lower values from the standard value observed for the carbonyl group due to the delocalization resulting from the presence of the carbonyl groups [27]. Thus, the IR spectra of the cyclic *exo*-ylides, (3,4-dihydro-1*H*-2-benzoselenin-2-io)methanides **18a**–**e** (Figure 12) exhibited strong IR absorptions, shifted **ca**. 100 cm^−1^ to a lower frequency than the typical absorption for the carbonyl and cyano groups. These shifts were explained by assuming that the ylidic carbanions are delocalized over the electron-withdrawing carbonyl and/or cyano groups [37]. From time to time, UV-VIS data is attached to the spectroscopic characteristics of a given selenonium ylide. The relevant data for several selenonium ylides, **9** [40], **12** [28], and **15a** [32], already mentioned in this review are shown in Figure 14.

In a single publication devoted to the use of circular dichroism spectroscopy as a tool in the structural determination of chiral selenonium ylides, the CD spectra of diastereomerically pure selenonium ylide (+)-**l5a** and diastereoisomerically enriched ylide (−)-**15b** were analyzed. This analysis showed that the Cotton effects at 266 and 251 nm are related to the absolute configuration around a stereogenic selenium in the ylide **15**. Thus, the *R* configuration in the selenonium ylide **15a** is related to a positive Cotton effect at 266 nm, and a negative Cotton effect at 251 nm, and consequently, the *S* configuration in the ylide **15b** is related to a negative Cotton effect at 266 nm, and a positive Cotton effect at 251 nm [32].

There is a single publication dedicated specifically to the use of mass spectrometry as a tool in the structural determination of selenonium ylides [41]. Among the ylides, for which fragmentation modes are discussed in this paper, there are compounds **9**–**11,** already mentioned in this manuscript. Moreover, the detailed analysis of the mass spectrum of the selenonium ylide **11** showed that the molecular ion at *m*/*z* = 332 has the greatest abundance, thus indicating its high stability. Other fragments were observed at *m*/*z* = 234, which corresponds to Ph_2_Se^+^, and at *m*/*z* = 157, which corresponds to C_6_H_6_Se^+^. The loss of one methyl group, which gave the peak with *m*/*z* = 317, was also observed [27]. Very recently, HRMS spectra were reported for a series of selenonium ylides **19a**–**f** [38].

**Figure 15:**
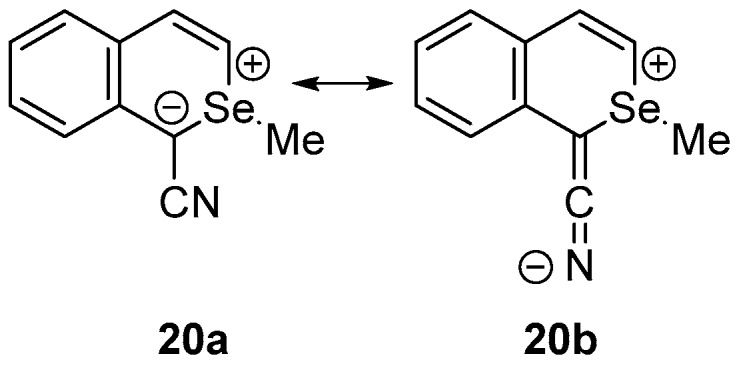
Stable selenabenzene **20**.

The various spectroscopic techniques, which were mentioned above, were applied to determine the structural features of selenabenzene derivatives, which formally can be considered as the specific group of selenonium ylides [41,42]. One such example is stable l-cyano-2-methyl-2-selenanaphthalene, **20** [43]. It was found that the IR absorption of its cyano group observed at 2130 cm^−1^ as a broad very strong band was shifted to the lower frequency than normal by about 100 cm^−1^. This was suggested as strong evidence that the selenanaphthalene **20** is stabilized by the contribution of the ketenimine resonance structure **20a** (Figure 15).

## 3. Synthesis

**Scheme 2:**
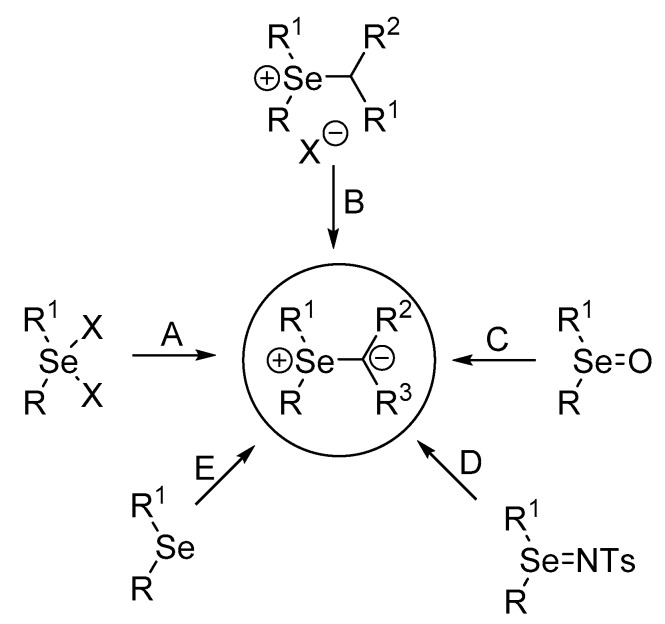
Major synthetic routs to selenonium ylides; (A) Reaction of dihaloselenuranes with activated methylene compounds in the presence of bases, (B) deprotonation of selenonium salts with bases, (C) reaction of selenoxides with activated methylene compounds or electron-deficient alkynes, (D) reaction of selenonium (*N-*arylsulfon)imides with activated methylene compounds, and (E) reaction of selenides with aryliodonium zwitterions or diazocompounds.

The major routes for the synthesis of selenonium ylides are as shown in Scheme 2.

### 3.1. Achiral and Racemic Compounds

**Scheme 3:**
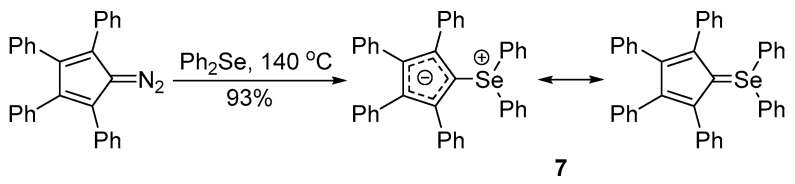
Synthesis of the first stable selenonium ylide **7**.

The synthesis of the first stable selenonium ylide was reported by Lloyd and Singer in 1967 [21]. It was based on the reaction of a carbene generated by the thermal decomposition of diazotetraphenylcyclopentadiene with diphenylselenide (Scheme 3). After a simple workup, selenonium ylide **7** was obtained in a 93% yield as air stable yellow crystals.

**Scheme 4:**
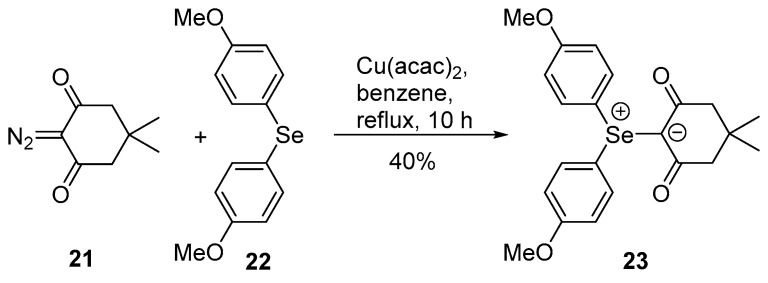
Synthesis of ylide **23** via the reaction of diazocompound **21** with selenide **22**.

The Cu(acac)_2_-promoted reaction of diazodimedone (**21**) with bis(4-methoxyphenyl)selenide (**22**) was successfully applied in the synthesis of stable ylide **23** (Scheme 4) [44].

**Scheme 5:**
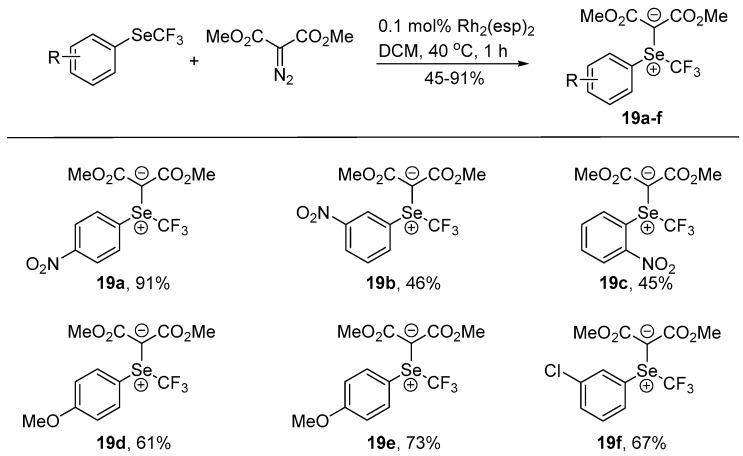
Preparation of Se-trifluoromethyl-substituted selenonium ylides **19a**–**f**.

Recently, an efficient and robust carbenoid version of the so-called “diazo approach” to the synthesis of stable trifluoromethyl(aryl) selenonium ylides was developed by Ge and Shen [38]. They found that the treatment of trifluoromethyl(aryl)selenides with dimethyl diazomalonate in the presence of a catalytic amount of a rhodium salt (Rh_2_(esp)_2_ bis[rhodium(α,α,α′,α′-tetramethyl-1,3-benzenepropionic acid)]) for 1 h at 40 °C afforded air- and moisture-insensitive crystalline selenonium ylides **19a**–**f** in 45–91% yields (Scheme 5).

**Scheme 6:**
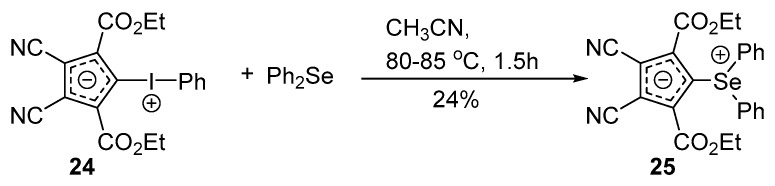
Synthesis of selenonium ylide **25**.

The displacement of an aryliodonium group from aryliodonium zwitterion **24** with diphenyl selenide was employed for the preparation of **25** [45]. After reflux for 1.5 h of a mixture of aryliodonium zwitterion **24** with diphenyl selenide in acetonitrile, stable selenonium ylide **25** was isolated in 24% (Scheme 6).

**Scheme 7:**
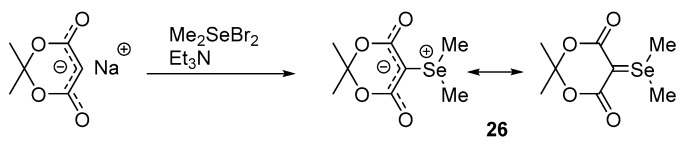
Synthesis of 5-dimethylselanylidene-2.2-dimethyl-1,3-dioxane-4,6-dione (**26**).

Selenonium ylides stabilized by two electron-withdrawing substituents on the carbanionic center were generated by the reaction of the corresponding dihalogenoselenuranes and activated methylene compounds. For the first time, this approach was applied as early as in 1971 when 5-dimethylselanylidene-2.2-dimethyl-1,3-dioxane-4,6-dione (**26**) was prepared by the reaction of dibromodimethylselenurane with one equivalent of sodium salt of Meldrum acid in the presence of triethylamine (Scheme 7) [46].

**Scheme 8:**
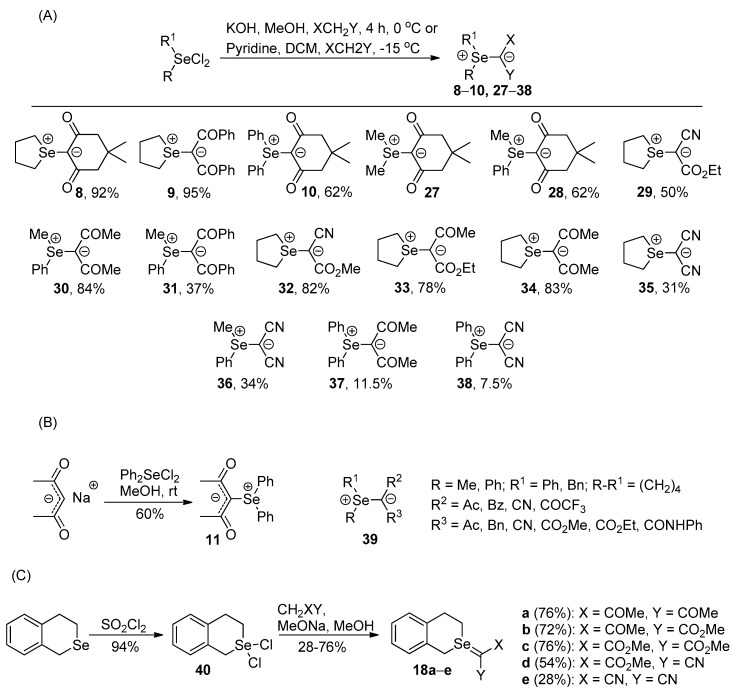
Selenonium ylides synthesized from dihalogenoselenuranes and activated methylene compounds. (**A**) from dimethyl, methylphenyl, diphenyl and selenophene dihalogenoselenouranes; (**B**) from methylphenyl, diphenyl and selenophene dichloroselenouranes; (**C**) from cyclic dichloroselenouranes **40**.

Such an approach to the synthesis of selenonium ylides was also independently developed by Russian chemists for the preparation of diphenylselenonium dimedoneylide **10** [47]. Based on this strategy, this group and a number of others synthesized later on several stable acyclic and cyclic selenonium ylides **27** [22], **8**–**10, 28**–**38** [26,40], **11** [27], **39** [48], and **18a**–**e** [37] containing two electron acceptor groups, such as carbonyl, alkoxycarbonyl, or nitryl, attached to the ylide carbon atom (Scheme 8).

**Scheme 9:**
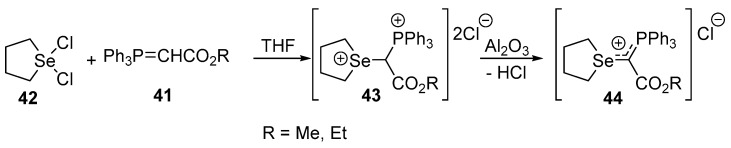
Synthesis of selenonium ylide salts **44**.

Expanding the above strategy, Magdesieva and Kyandzhetsian worked out in 1973 a simple two-step protocol for the preparation of stable selenonium ylide salts [49,50]. It encompassed the treatment of stabilized phosphonium ylides **41** with dichloroselenurane **42**, affording double salts **43**, which in the presence of Al_2_O_3_ were converted into the white solid ylide salts **44** (Scheme 9).

**Scheme 10:**
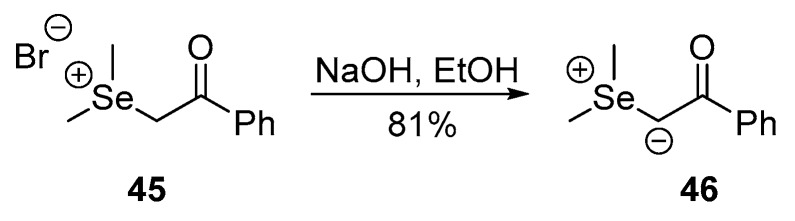
Synthesis of ylide **46** via deprotonation of selenium salt **45**.

Stable selenonium ylides have also been prepared by deprotonation of the corresponding selenonium salts. This approach was first applied by Lotz and Gosselck in the synthesis of acyclic ylide, stabilized by one benzoyl group, **46**, which was obtained from the corresponding selenonium bromide **45** upon treatment with sodium hydroxide (Scheme 10) [51].

**Scheme 11:**
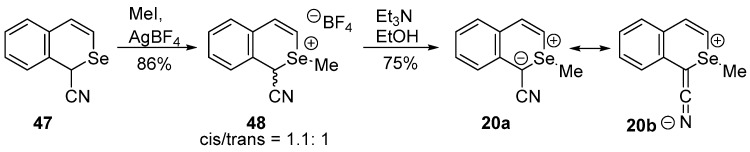
Preparation of selenabenzene derivative **20**.

The same method was practiced for the preparation of a stable selenabenzene derivative with an electron-withdrawing group and the ylidic C-Se bond constituting a part of a cyclic conjugated six-electron system. In 1990, Hori and co-workers reported that methylation of 1-cyanoisoselenochromene (**47**) with methyl iodide and silver tetrafluoroborate followed by the reaction of diastereoisomeric 1-cyano-2-methylisoselenochromeniumte trafluoroborates (**48)** with triethylamine in ethanol gave in good yield l-cyano-2-methyl-2-selenanaphthalene **20** as orange needles (Scheme 11) [43].

**Scheme 12:**
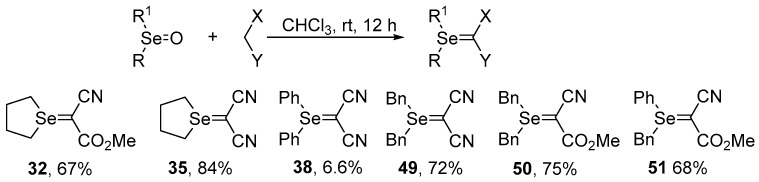
Synthesis of selenonium ylides **32**, **35**, **38**, **49**–**51** from dialkyl or arylalkyl selenoxides and activated methylene compounds.

**Scheme 13:**
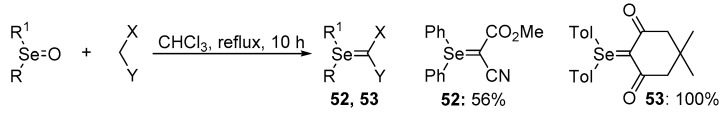
Synthesis of selenonium ylides **52** and **53** from diaryl selenoxides.

Another class of compounds that is widely used for the preparation of selenonium ylides are selenoxides. In 1975, Tamagaki and Sakaki reported a very simple and convenient procedure for the preparation of selenonium ylides involving the reaction of dialkyl or arylalkyl selenoxides with an equimolar amount of malononitrile or methyl cyanoacetate in chloroform at ambient temperature and with or without the addition of desiccating agents (Scheme 12) [52]. They found that the reaction of diphenyl selenoxide with malononitrile afforded a 53.4% yield of the reduction product, i.e., diphenyl selenide as the main product with a poor amount of ylide (**38**: 6.6%), whereas in that with methyl cyanoacetate, only the reduction product was detected. As it was reported a few years later, diphenyl and ditolyl selenoxides were effectively converted into the ylides **52** and **53** by refluxing with an equimolar amount of cyanoacetate or dimedone in CHCl_3_, respectively (Scheme 13) [53].

**Scheme 14:**
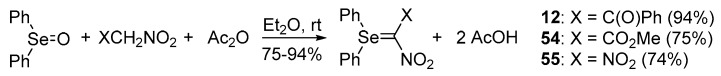
Synthesis of selenonium nitroylides **12** and **54**,**55** from diphenyl selenoxide.

In 1978, Shevetev and coworkers published an easy procedure for the preparation of selenonium ylides containing the nitro group attached directly to the ylide carbon atom [54]. Selenonium nitroylides were obtained in high yields (75–94%) by reacting equimolar amounts of aliphatic nitro compound with diphenylselenium oxide in the presence of acetic anhydride (Scheme 14). Selenonium nitroylides **12** and **54** are fairly stable in the crystalline form at room temperature, while **55** undergoes gradual decomposition (to the extent of 40% in 16 h at 20 °C). All compounds are stable as solutions in dimethyl sulfoxide (DMSO )and CH_3_CN. Unlike the sulfonium analogs, the selenonium nitroylides are easily hydrolyzed by water to give diphenyl selenoxide and the corresponding nitroalkane.

**Scheme 15:**
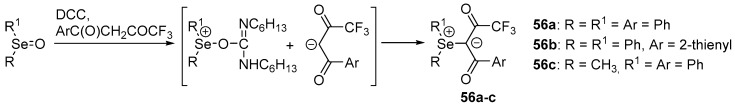
DCC-promoted synthesis of ylides **56** from selenoxides.

Condensation of stable selenoxides with activated methylene compounds promoted by the addition of dicyclohexylcarbodiimide (DCC) was employed by Magdesieva and coworkers for the preparation of selenonium ylides **56a**–**c** with a trifluoroacetyl group attached to the ylidic carbon atom (Scheme 15) [55].

**Scheme 16:**
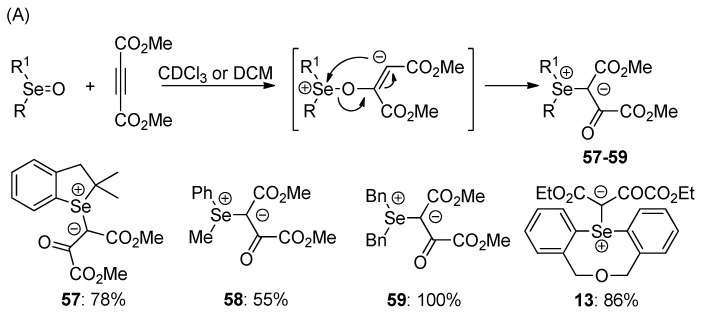

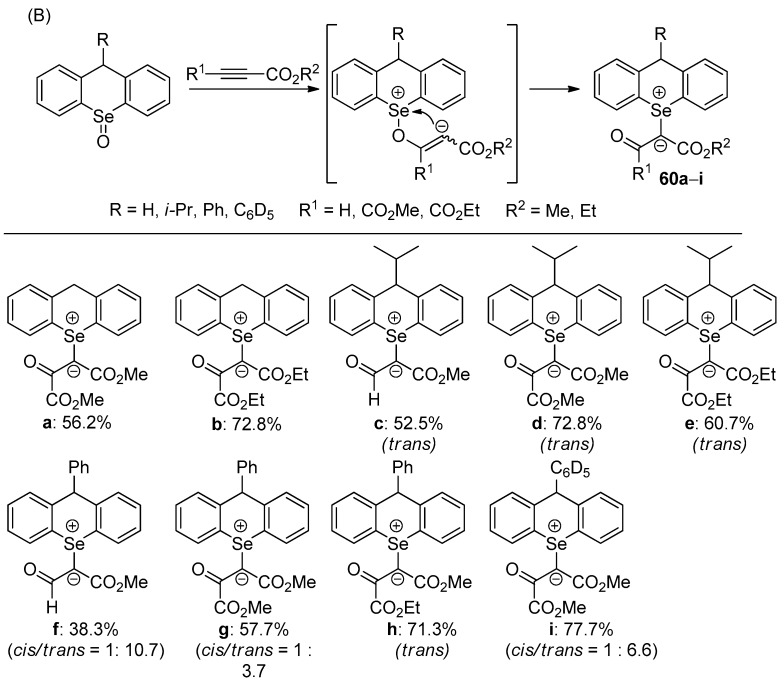
Synthesis of selenonium ylides **60** via the addition of selenoxides to acetylenic compounds. (**A**) from the variety of selenoxides and diacylacetylene; (**B**) from 9-substituted-9*H*-selenoxanthene 10-oxide.

Beside active methylene compounds, electron-deficient alkynes also react with selenoxides to give selenonium ylides. Usually, these reactions proceed smoothly even at ambient temperature and lead to the selenonium ylides in good yields. Based on this method, several stable selenonium ylides **57**, **58** [56,57], **59** [58], **13** [29], and **60a**–**i** [59] were prepared (Scheme 16).

**Scheme 17:**
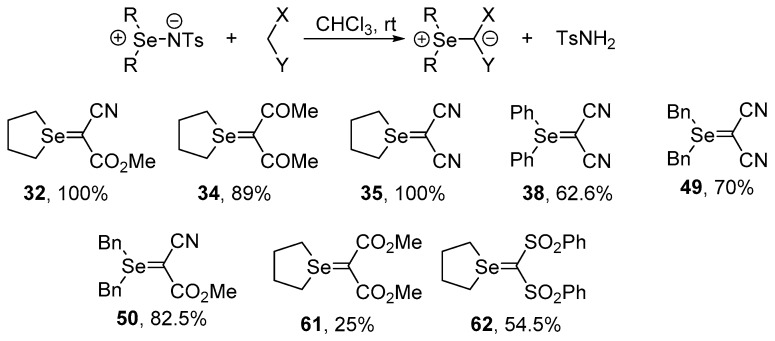
Synthesis of selenonium ylides **32**, **34**, **35**, **38**, **49**, **50** and **61**, **62** from selenonium *N-p*-toluenesulfonimides.

As it was demonstrated by Tamagaki and Sasaki, selenonium ylides can also be easily prepared from selenonium sulfonimides [52]. Thus, treatment of selenonium *N*-*p*-toluenesulfonimides with activated methylene compounds afforded the corresponding stable selenonium ylides even at room temperature (Scheme 17).

**Scheme 18:**
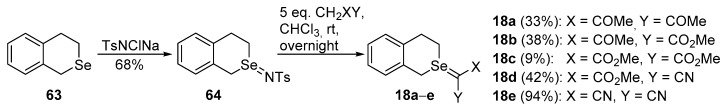
Preparation of selenonium ylides **18** from selenonium *N-p*-toluenesulfonimide **64**.

This method was successfully applied in the synthesis of stable cyclic selenonium *exo*-ylides **18**, which were obtained in a two-step process from 3,4-dihydro-1*H*-2-benzoselenin (**63**) (Scheme 18) [37]. In the first step, selenide **63** was reacted with chloramine T trihydrate to give *N*-*p*-tolueneselenimide **64**, which in the next step was treated with five equivalents of activated methylene compounds, such as acetylacetone, methyl acetoacetate, dimethyl malonate, methyl cyanoacetate, and malononitrile, to give selenonium ylides **18a**–**e**, respectively.

### 3.2. Optically Active Selenonium Ylides

**Scheme 19:**
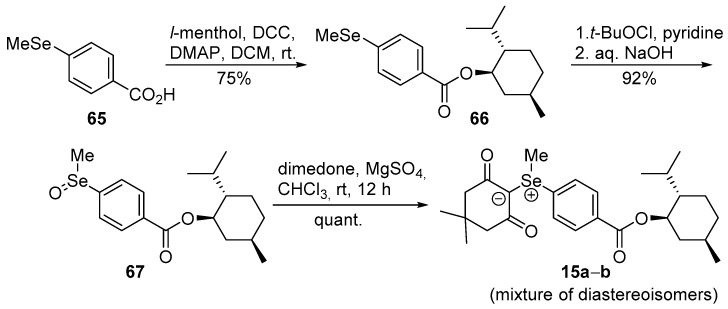
Synthesis of optically active diastereoisomeric selenonium ylides **15a**,**b**.

The first optically active diasteoisomerically pure selenonium ylide was reported by Kamigata and co-workers in 1991 [32,60]. According to the developed protocol, the esterification reaction of *p*-methylselenobenzoic acid (**65**) with *l*-menthol followed by the oxidation of ester **66** with *t*-butyl hypochlorite afforded selenoxide **67** in 69%, which upon treatment with dimedone gave a diastereomeric mixture of selenonium ylides **15a,b** in a quantitative yield (Scheme 19). Diastereoisomerically pure ylide (+)-**15a** was isolated as stable crystals by fractional recrystallization of the diastereomeric mixture **15a,b** from hexane-diethyl ether. Diastereoisomer (−)-**15b**, obtained from a mother liquid, had only 27% diastereomeric purity.

**Scheme 20:**
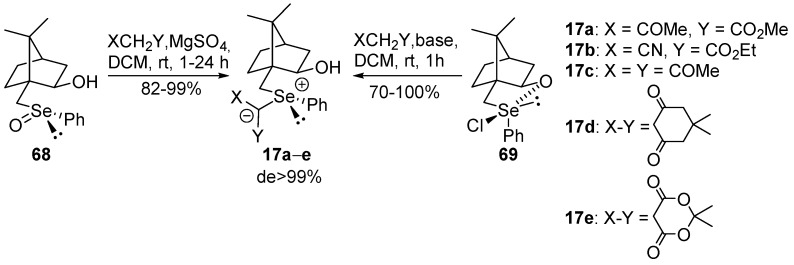
Reaction of optically active selenoxide **68** and chloroselenurane **69** with activated methylene compounds.

A few years later, another Japanese group reported the synthesis of a series of diastereoisomerically pure selenonium ylides **17** by using the 2-*exo*-hydroxy-10-bornyl group as a chiral auxiliary [34]. They found that the reaction of optically active (*R*_Se_)-selenoxide **68** and nucleophilic substitution reaction of (*R*_Se_)-chloroselenurane **69** with active methylene compounds proceeded with retention of the configuration, affording diastereoisomerically and enantiomerically pure selenonium ylides **17a**–**e** in high yields (Scheme 20).

## 4. Synthetic Applications

Selenium-containing compounds are highly valuable reagents and catalysts with widespread application in the organic chemistry (i.e., synthesis of complex natural products, pharmaceutical ingredients, new materials, and as a tool for the introduction of new functional groups). Due to the specific chemical reactivity, it could be expected that selenonium ylides should serve as useful synthetic reagents. Until now, their synthetic applicability, mainly to induce several types of C–C bond formation reactions, is still limited. In this part of the review, we comprehensively discuss such applications to the cyclopropanation, epoxidation reactions, synthesis of α,β-unsaturated ketones, and sigmatropic rearrangements.

### 4.1. Cyclopropanation Reactions

**Scheme 21:**
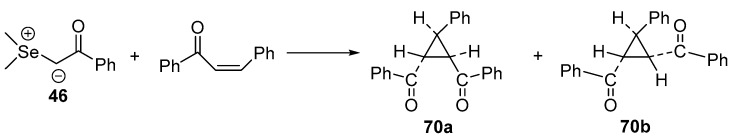
Synthesis of cyclopropanes **70a,b**.

In 1973, Lotz and Gossleck described the use of selenonium ylides in the preparation of cyclopropanes [51]. The ylide **46** obtained from the appropriate selenonium bromide reacted with benzalacetophenone, giving *cis*-phenyl-dibenzoil-cyclopropane **70a** as the main product (Scheme 21).

**Scheme 22:**
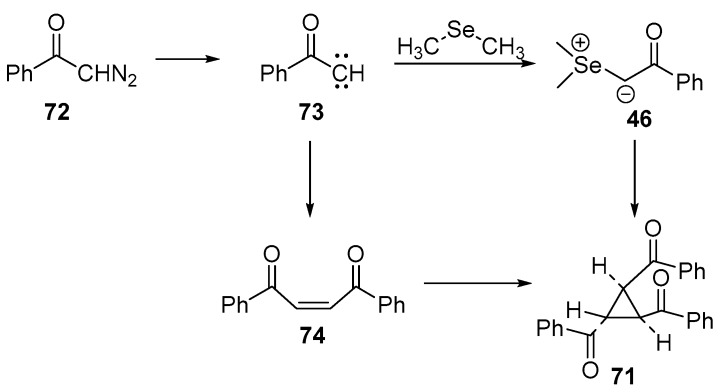
The formation of *trans*-tribenzoilcyclopropane **71**.

The reaction carried out with the isolated ylide **46** and benzalacetophenone afforded the *trans* isomer **70b**. Moreover, the use of a slight excess of sodium hydroxide solution and benzalacetophenone led to the mixture of *cis*-*trans* cyclopropanes. The termal and photolytic decomposition of selenonium ylide **46** afforded *trans*-tribenzoilcyclopropane **71** formed by the reaction sequence shown in Scheme 22. The decomposition of benzoildiazomethane **72** led to the carbene **73**, which dimerizes and forms dibenzoiloethylene **74**. In the presence of dimethyl selenide, which acts as a carbene catcher, the formed ylide **46** reacts with the activated double bond of **74** to form cyclopropane **71**.

**Scheme 23:**
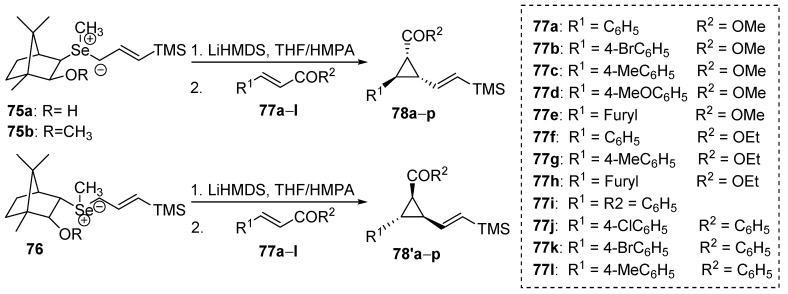
Synthesis of *cis*-trisubstituted cyclopropanes **78** and **78** from *exo*- and *endo*-camphor selenonium ylides **75a**,**b** and **76**.

The first application of the optically active selenonium ylides in asymmetric cyclopropanation was reported by Huang’s group [61]. At first, they conducted the reactions of *exo*- and *endo-*camphor selenonium ylides **75a,b** and **76** with various α,β-unsaturated carbonyl compound **77a**–**l** (Scheme 23). For both *exo*-selenonium ylides **75a** and **75b**, the corresponding *cis*-(1*R*,2*R*,3*R*)-trisubstituted cyclopropanes **78** were obtained in good to excellent yields, high diastereoselectivities, and good to excellent enantioselectivities (Table 1).

Similarly, for the reaction carried out with *endo*-selenonium ylide **76**, the asymmetric cyclopropanation reaction led to the *cis*-(1*S*,2*S*,3*S*)-trisubstituted cyclopropanes **78′** in good yield, with excellent diastereoselectivities and enantioselectivities (Table 2).

**Scheme 24:**
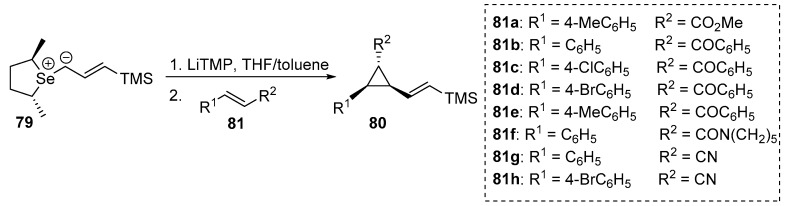
Synthesis of *trans*-(1*R*,2*R*,3*S*)-trisubstituted cyclopropanes **80** from selenonium ylide **79**.

In the same paper, the asymmetric cyclopropanation via *C_2_*-symmetric selenonium ylide **79** was described (Scheme 24). The corresponding *trans*-(1*R*,2*R*,3*S*)-cyclopropanes **80** were obtained in good yield, high diastereoselecitvities (d.r. 95:5 > 99:1), and excellent enantioselectivities (up to 99% *ee*) (Table 3).

In 2014, Midura and coworkers reported the asymmetric cyclopropanation of vinyl phosphonates using optically active selenonium ylides derived from (−)-menthol and (+)-limonene [62]. The asymmetric cyclopropanation of vinyl phosphonates **82** and **83** with benzyl terpenyl selenonium salts **84**–**86** occurred in moderate to good yields (20–54%), while the diastereoselectivity was observed only in the case of vinyl phosphonate **83**. Analysis of the enantiomers showed that their ratio depends on the chiral substituent bonded to the selenonium salts used in the reaction. Reaction of vinyl phosphinates **83** with menthol-derived selenonium salts **84** and **86** led to a cyclopropanation product with enantioselectivity of approximately 27% (Table 4, entry 1 and 4). The application of isoselenocineole-derived selenonium salt **85** led to enantioselectivities in the range of 88–92% (Table 4, entry 2).

The authors also performed the cyclopropanation reaction using the corresponding selenonium salt **89,** which contain the ethyl acetate substituent. The reactions afforded the *trans*- and *cis*-cyclopropanes **90** and **91** in good yield (75–86%), with moderate diastereoselectivity and excellent enantioselectivity (up to 99:1) (Table 5).

### 4.2. Epoxidation Reactions

**Scheme 25:**
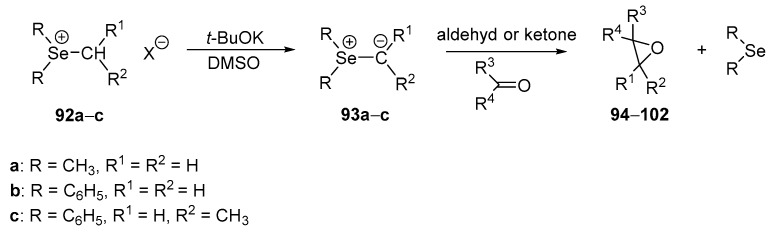
Synthesis of epoxides **94**–**102** from selenonium salts **92a**–**c**.

In 1974, Krief and his group reported the first example of the reaction of selenium ylides with carbonyl compounds, which provides epoxides [63]. Selenonium ylides **93a**–**c** generated in situ from selenium salts **92a**–**c** using potassium *tert*-butoxide as a base were found to react with aldehydes or ketones to form the corresponding epoxides **94**–**102** in good to high yields (50–90%) (Scheme 25, Table 6).

It is worth noting that in the reaction of selenonium salts with enolizable carbonyl compounds (i.e., heptanal, cyclohexanone, methyl ethyl ketone, and acetophenone), the corresponding epoxides were not formed. Such a result is caused by the formation of an acetophenone anion, which is subsequently methylated by the selenonium salt. The generation of the selenonium ylides prior to the addition of acetophenone allowed the avoidance of the methylation reaction.

**Scheme 26:**
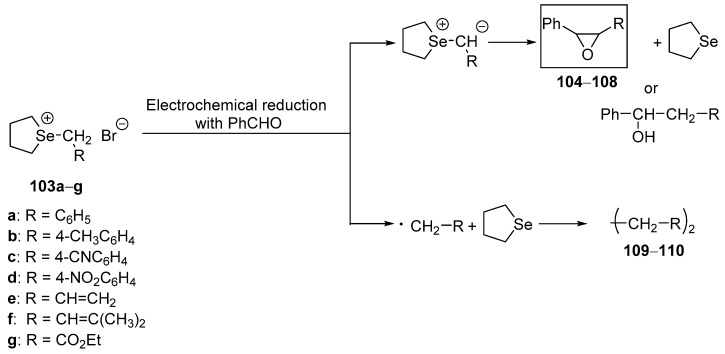
Electrochemical reduction of substituted methyltetrahydroselenonium bromide **103a**–**g** in the presence of benzaldehyde.

**Scheme 27:**
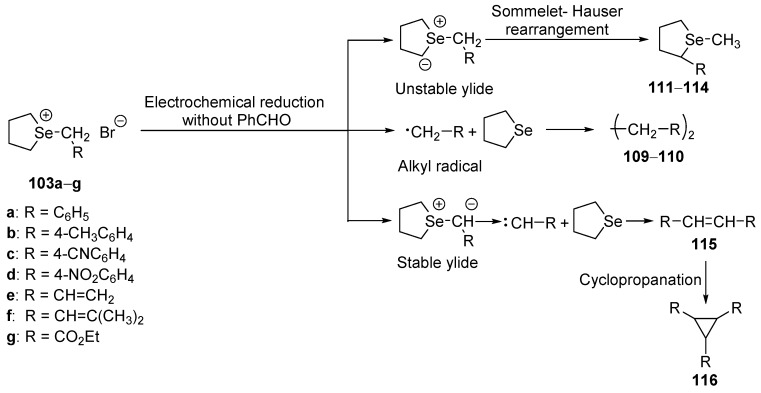
Electrochemical reduction of selenonium salts **103a**–**g** without benzaldehyde.

Morihara’s group reported the generation of the selenonium ylides by electrochemical reduction of the cyclic five-membered selenonium salts **103a**–**g** carried out in the presence or without benzaldehyde (Scheme 26 and Scheme 27) [64].

When electrochemical reduction of selenonium salts **103a**–**g** was carried out in the presence of acetophenone, the main reaction was epoxidation (products **104**–**108**); only in two cases, a product of a radical coupling (**109**–**110**) was formed. In most cases, moderate to good yields were observed (Table 7).

For electrochemical reduction carried out in the absence of benzaldehyde, the reaction course depends on the *R* substituent in the selenonium salt (Scheme 27). When the reactions were conducted on selenonium salts bearing a benzyl or allyl substituent, the products of the [2,3]-sigmatropic rearrangement (**111**–**114**) or radical coupling (**109**–**110**) were formed. In the case of a selenonium salt bearing a carbonyl group, formation of carbene led to olefins **115** or cyclopropanes **116** as products (Table 8).

**Scheme 28:**
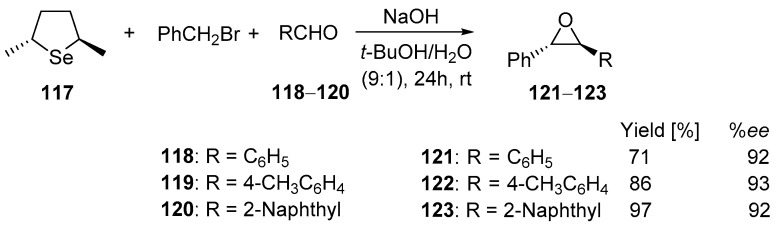
Asymmetric epoxidation of aldehydes by the reaction with selenonium ylides generated in situ from selenolane **117**.

In 2001, Metzner and coworkers reported the first application of an optically active selenonium ylide in the asymmetric epoxidation reaction [65]. The appropriate enantioenriched selenonium ylide was generated by the addition of benzyl bromide to C_2_-symmetric (2*R*,5*R*)-2,5-dimethylselenolane **117** in the presence of NaOH. Its reaction with a variety of aldehydes **118**–**125** led to the corresponding epoxides. Thus, the reaction of aldehydes **118**–**120** with the stoichiometric amount of selenide **117** afforded the corresponding epoxides **126**–**128** in the yield from 71% to 97% and with excellent enantiomeric excesses (92–93%) (Scheme 28).

**Scheme 29:**
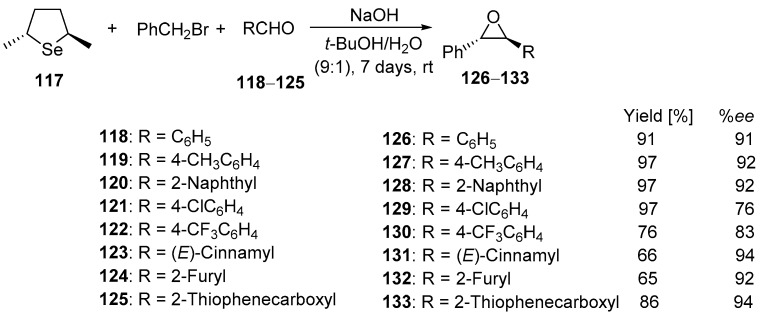
Catalytic asymmetric epoxidation of aldehydes in the presence of a catalytic amount of selenolane **117**.

In turn, the use of a catalytic amount of selenolane **117** (20% mol) in reaction with aldehydes **118**–**125** at ambient temperature led to the corresponding epoxides in good to excellent yields (65–97%). For more reactive heteroaromatic aldehydes (**124** and **125**), the reaction time was optimized to 4 h. In most cases, enantiomeric excesses were in the range 91–94%, except for aldehydes **121** and **122**, which bear electron-withdrawing groups (Scheme 29).

**Scheme 30:**
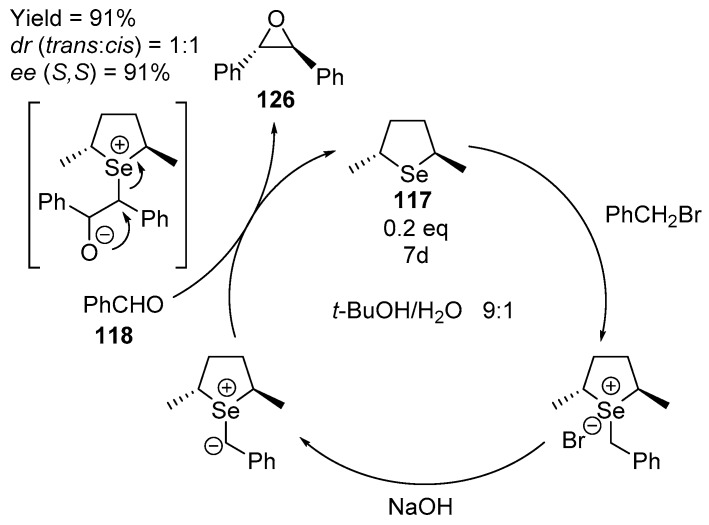
Mechanism of formation epoxides in the reaction of the selenolane **117**/benzylbromide system with aldehydes.

Four years later, Metzner’s group also proved that (2*R*,5*R*)-2,5-dimethylselenolane (**117**) is an efficient catalyst for the benzylidenation of aromatic aldehydes [66]. The authors noted that as compared to the sulfur analogues, the selennium-based system leads to enhanced reactivity and higher asymmetric induction, with the same absolute configuration. The reaction of selenolane **117** with benzaldehyde **118** led to the epoxide **126**, which was formed, surprisingly, with a lack of diastereoselectivity and with an enantiomeric excess higher than 90%. The authors explained [67,68] that the formation of equal amounts of *trans* and *cis* diastereomers is caused by a reversible betaine formation, leading to the *cis* epoxide, which is formed from the betaine *syn* conformer (Scheme 30).

**Scheme 31:**
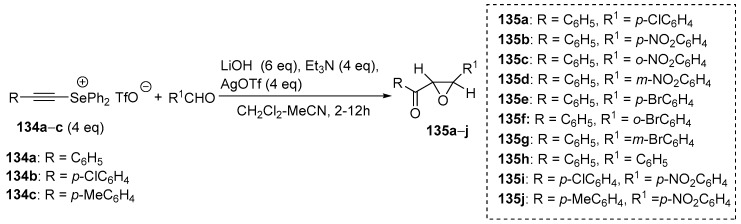
Reaction of ketoselenonium salts **134a**–**c** with aromatic aldehydes.

Watanabe and Kataoka described the use of ketodiphenylselenonium ylides generated from the corresponding alkynylselenonium salt for the synthesis of oxiranylketones [69]. The reaction of alkynylselenonium salt **134a**–**c** with various aromatic aldehydes in the presence of LiOH, silver triflate, and triethylamine gave oxiranylketones **135a**–**j** just as a *trans*-isomer in moderate to good yields (40–92%) (Scheme 31, Table 9).

**Scheme 32:**
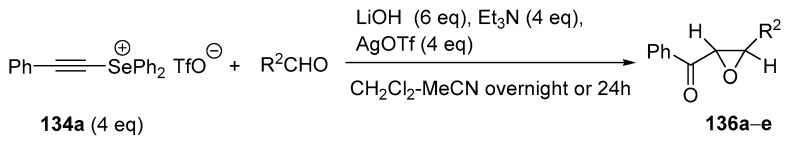
Reactions of ketoselenonium ylide **134a** with aliphatic aldehydes.

In the case of aliphatic aldehydes, the desired oxiranylketones **136a**–**e** were formed in moderate yields (18–54%) under the same reaction conditions (Scheme 32, Table 10).

**Scheme 33:**
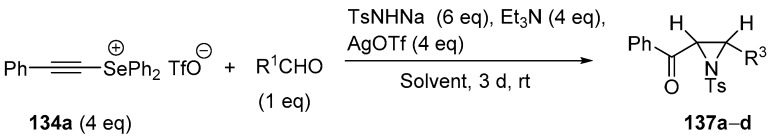
Reactions of alkynylselenonium salt **134a** with aromatic aldehydes and TsNHNa.

In turn, the reaction of alkynylselenonium salt **134a** with aromatic aldehydes in the presence of sodium *p*-toluenesulfonamide instead of LiOH led to the corresponding benzoyl aziridine derivatives **137a**–**d** in moderate yields (Scheme 33, Table 11). The comparison of the coupling constants values with literature data indicates that these isomers have the *cis* geometry (*J*_HH_ the methine protons on the aziridine ring is 7–8 Hz) [70].

**Scheme 34:**
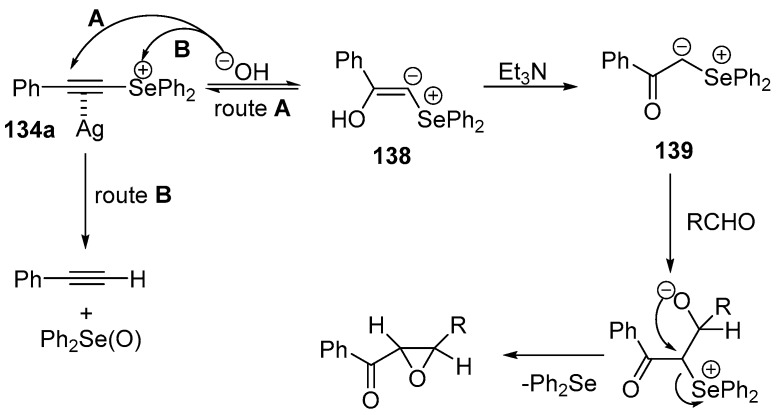
Plausible mechanism of the reactions of alkynylselenonium salt **134a** with an aldehyde and hydroxide ion in the presence of silver cation and Et_3_N.

Scheme 34 shows the plausible mechanism for the reactions of alkynylselenonium salt **134a** with aldehydes and the hydroxyl ion in the presence of a silver salt and triethylamine (Scheme 34). In the first step, the triple bond of the selenonium salt is activated by silver cation, and the hydroxyl ion attacks the β-carbon atom to form the vinyl ylide **138**. In the presence of Et_3_N, the ylide **138** is transformed into the ketodiphenylselenonium ylide **139**, which reacts with an aldehyde to form appropriate oxiranylketones (route A). In turn, diphenyl selenoxide is formed by the attack of hydroxyl ion on a selenonium cation without activation of a triple bond by a silver ion (route B) [71,72].

### 4.3. Synthesis of α,β-Unsaturated Ketones

**Scheme 35:**
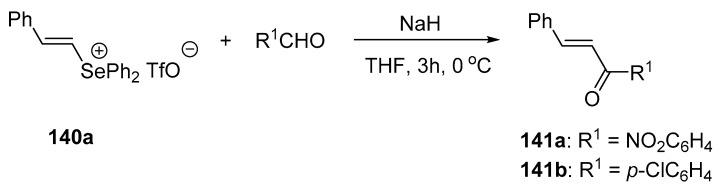
Synthesis of α,β-unsaturated ketones **141a,b** from a vinylselenonium salt.

Watanabe and Kataokan also reported the first application of vinylselenonium ylide, generated in situ from (*Z*)-vinylselenonium salts **140a** in the presence of sodium or potassium hydride as a base, in the synthesis of α,β-unsaturated ketones [73]. When the reactions of selenonium salt **140a** were carried out with sodium hydride as a base, the best results were obtained for the reaction with *p*-nitrobenzaldehyde, and the corresponding *p*-nitrophenyl styryl ketone **141a** was obtained in 65%. The reaction with *p*-chlorobenzaldehyde led to ketone **141b** only in an 11% yield. The use of 2 equiv. of sodium hydride instead of 1.3 equiv. increased the yield of the product to 58% (Scheme 35). In turn, the reactions with benzaldehyde or *p*-tolualdehyde afforded complex mixtures, including unreacted aldehydes, while the desired α,β-unsaturated ketones were not isolated.

**Scheme 36:**
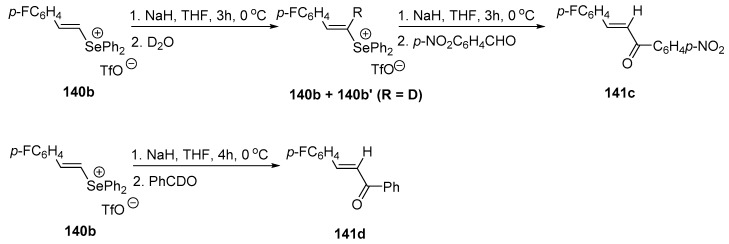
Reaction of vinylselenonium salt **140b** with D_2_O and PhCDO. Confirmation of the selenonium ylide formation.

The formation of the vinylselenonium ylides in this reaction was confirmed by the experiment carried out on vinylselenonium salt **140b** with 1 equiv. of sodium hydride in tetrahydrofurane (THF) at 0 °C for 3 h without the aldehyde, followed by treatment with D_2_O. The obtained mixture of **140b** and **140b′** while treated with sodium hydride in the presence of *p*-nitrobenzaldehyde under the same conditions as for the reaction of **140a** gave the compound **141c** in a 49% yield. In turn, the reaction of vinylselenonium salt **140b** with deuterated benzaldehyde led to the compound **141d** in a 14% yield (Scheme 36). In both cases, the obtained ketones did not contain a deuterium atom.

When the reactions of vinylselenonium salt **140a** with aromatic aldehydes were carried out in the presence of potassium hydride as a base, the desired α,β-unsaturated ketones **141a**,**b** and **141e**–**g** were obtained in higher yields than in the case of the reactions carried out with sodium hydride (Table 12). Better yields were obtained when aldehydes with an electron-withdrawing group were used in the reaction instead of those with an electron-donating group.

**Scheme 37:**
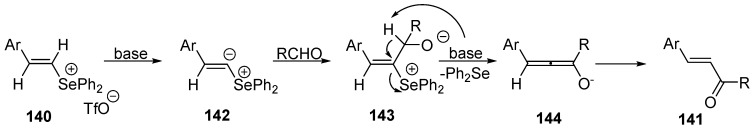
The plausible mechanism of the reactions of vinylselenonium salts with aldehydes.

The plausible mechanism for the reactions of vinylselenonium salts with aldehydes is shown in Scheme 37. The reaction of vinylselenonium salt **140** with a base led to the formation of vinylselenonium ylide **142**, which in the next step reacted with an aldehyde and gave betaine **143**. Deprotonation of betaine **143** and β-elimination of diphenylselenide led to the adduct **144**, which isomerized to the corresponding α,β-unsaturated ketone **141,** probably via a hydride transfer.

### 4.4. Sigmatropic Rearrangements

**Scheme 38:**
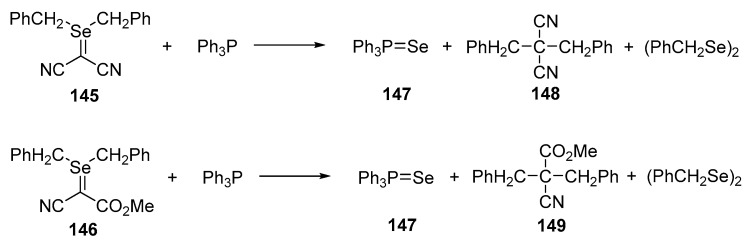
Reaction of dibenzylselenonium dicyanomethylide **145** and dibenzylselenonium cyanomethoxycarbonylmethylide **146** with triphenylphosphine.

The reactions of dibenzylselenonium dicyanomethylide **145** and dibenzylselenonium cyanomethoxycarbonylmethylide **146** with triphenylphosphine were examined by Tamagaka and coworkers [74]. They found that the reaction of dibenzylselenonium dicyanomethylide **145** carried out at room temperature led to the mixture of products: Triphenylphosphine selenide **147** (87%), 2-benzyl-2-cyano-3-phenylpropanenitrile **148** (53%) and a small amount of dibenzyl diselenide (11%). The treatment of dibenzylselenonium cyanomethoxycarbonylmethylide **146** with triphenylphosphine afforded methyl 2-benzyl-2-cyano-3-phenylpropanoate **149**, triphenylphosphine selenide **147**, and dibenzyl diselenide in 29%, 49%, and 28% yields, respectively. In both cases, products obtained in the reactions were formed via the deselenization reaction and no dibenzyl selenide was observed (Scheme 38).

**Scheme 39:**
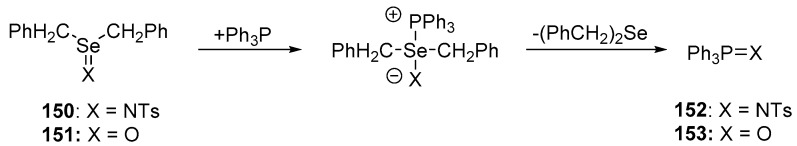
The reaction of dibenzylselenonium *N*-tosylimide **150** with triphenylphosphine.

On the contrary, the reaction of dibenzylselenonium *N*-tosylimide **150** with triphenylphosphine at room temperature led to the formation of dibenzyl selenide (81%) as the main product, triphenyl *N*-tosylphosphimine **152** (54%), and only a trace of triphenylphosphine selenide (6%) (Scheme 39). The isolation of the phosphine selenide indicates that the selenonium imide **150** also undergoes deselenization but as a result of a minor process. In turn, the reaction of dibenzyl selenoxide with triphenylphosphine was found to be much more facile and led quantitatively to dibenzyl selenide and triphenylphosphine oxide **153**. No deselenization products were observed. Therefore, the reactivity order for the reduction into dibenzyl selenide is selenoxide > selenonium imide > selenonium ylide.

It was found that the addition of acetic acid or water to the reaction system changes the product distribution and reaction modes. The reaction of compounds **145** and **146** under these conditions gave dibenzyl diselenides as the main product. Meanwhile, the corresponding selenonium imide **150** and selenoxide **151** afforded dibenzyl selenide.

**Scheme 40:**
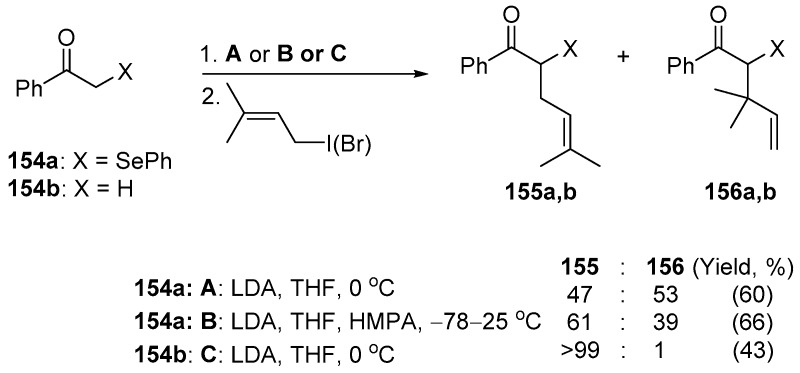
Reaction of α-(phenylseleno)acetophenone **154a** with prenyl iodide or bromide.

Reich and Cohen reported that the seleno-substituted ketone enolates can undergo an alkylation at the heteroatom [75]. Treatment of the appropriate enolate of α-(phenylseleno)acetophenone **154a** with prenyl iodide or bromide led to the alkylation product **155a** and unexpected product **156a** (Scheme 40).

**Figure 16:**
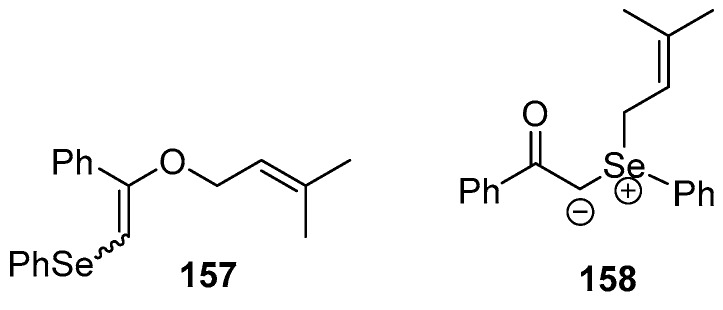
Enolate **157** and ylide **158**.

The formation of compound **156a** may result from two reaction pathways. The first one is the *O*-alkylation of enolate to give **157**, followed by the [3,3]-sigmatropic rearrangement, while the second one assumes alkylation at the selenium atom to form the ylide **158** and the subsequent [2,3]-sigmatropic rearrangement (Figure 16).

**Scheme 41:**
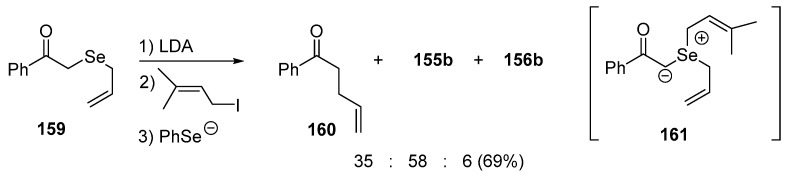
Alkylation of α-allylselenoacetophenone (**159**).

To confirm the ylide mechanism, the alkylation of α-allylselenoacetophenone **159** was performed. One of the reaction products was *C*-allyl product **160**, which must be formed by the [2,3]-sigmatropic rearrangement of the intermediate **161** capable of allyl migration [76] (Scheme 41).

**Scheme 42:**
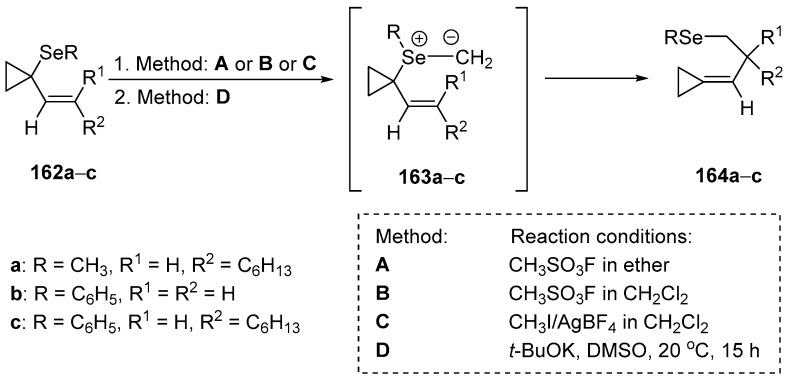
Synthesis of alkylidenecyclopropanes **164a*–*c**.

Krief’s group reported the [2,3]-sigmatropic rearrangement of allylic selenonium ylides and the application of the rearrangement to the synthesis of functionalized alkylidene cyclopropanes [77]. The alkylation reaction (method A, B, or C) of the corresponding 1-seleno-l-vinyl cyclopropanes **162a**–**c** led to the selenonium salts, which in the reaction with *t*-BuOK afforded appropriate selenonium ylide as an intermediate, which subsequently rearranged to alkylidenecyclopropanes **164a**–**c** (Scheme 42, Table 13).

**Scheme 43:**
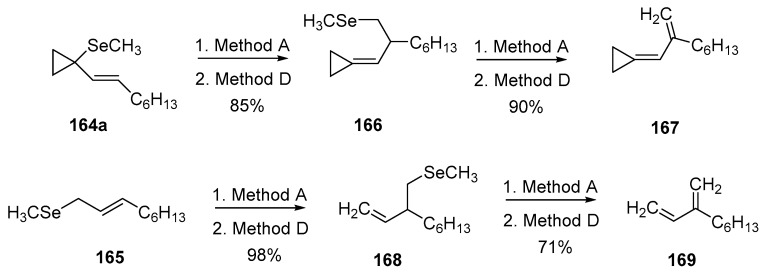
Synthesis of dienes **167** and **169**.

It was found that the homoallyl selenides **164a** and **165** are good precursors of dienes. The alkylation reaction of the selenium atom (method A) followed by treatment with *t*-BuOK/DMSO (method D) led to the dienes **167** and **169** by an elimination reaction in the regioselective way (Scheme 43).

**Scheme 44:**
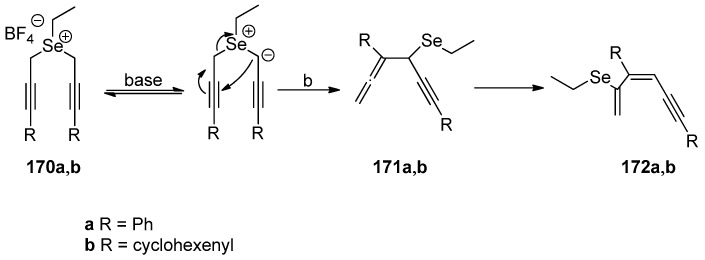
Base-catalyzed [2,3]-sigmatropic rearrangement of bis-γ-substituted bridged propargylic systems.

Braverman and coworkers reported a base-catalyzed [2,3]-sigmatropic rearrangement of bis-γ-substituted propargylic selenonium salts [78]. The reaction of ethyl bis-γ-cyclohexenylpropargyl selenium tetrafluoroborate **170a,b** with 1,8-diazabicyklo(5.4.0)undek-7-en (DBU) led to the appropriate selenonium ylide, which underwent a spontaneous [2,3]-sigmatropic rearrangement and gave the selenonium derivatives **171a,b**. The obtained compounds underwent a subsequent [1,3] shift to the appropriate dienynes **172a,b** (Scheme 44).

**Scheme 45:**
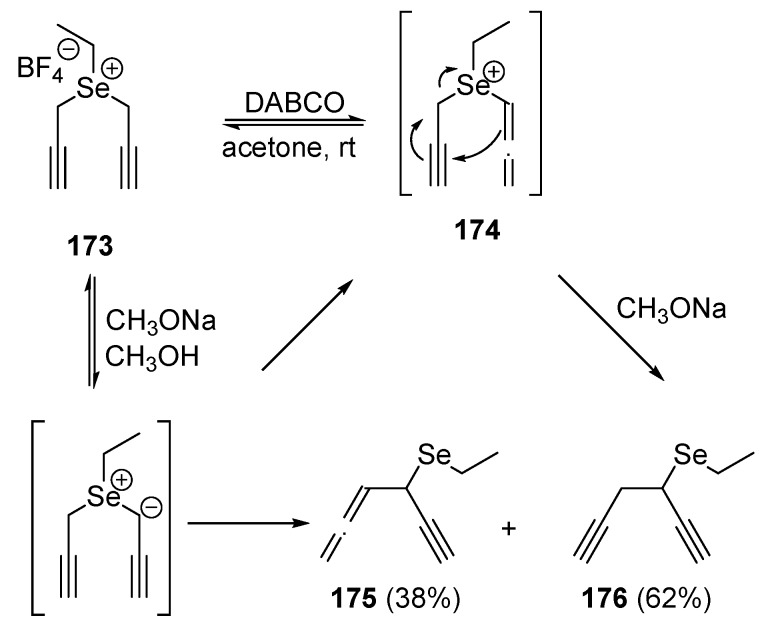
Rearrangements of unsubstituted bridged propargylic systems **173**.

Similarly, the selenonium salt **173** in the presence of CH_3_ONa undergoes [2,3]-sigmatropic rearrangements leading to the corresponding selenides **175** and **176**. Using weaker bases like 1,4-diazabicyclo[2.2.2]octane (DABCO) leads to mixtures of S_N_2 products (Scheme 45). This result indicates the relatively high sensitivity of selenonium salts to nucleophilic displacement with respect to the corresponding sulfonium salts, which may be explained by the relatively higher polarizability of the selenium atom as well as the weaker Se-C bond [37].

**Scheme 46:**
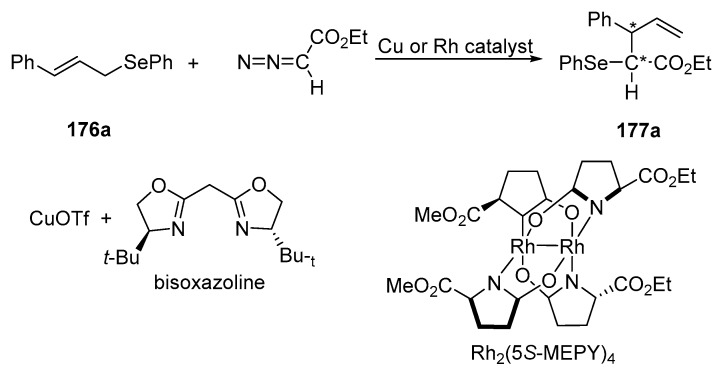
Reaction of (*E*)-cinnamyl phenyl selenides **176** with ethyl diazoacetate in the presence of a catalytic amount of copper or rhodium catalyst.

Uemura and coworkers described the first example of enantioselective addition of the carbenoid derived from ethyl diazoacetate to chalcogen atoms of aryl cinnamyl chalcogenides, which proceed via a [2,3]-sigmatropic rearrangement of the chalcogen ylide [79]. The reaction of (*E*)-cinnamyl aryl selenides **176a**–**c** with ethyl diazoacetate in the presence of a catalytic amount of copper or rhodium catalyst led to the mixture of diastereomeric ethyl 2-arylchalcogeno-3-phenylpent-4-enoates **177a**–**c**. When the reaction was carried out on (*E*)-cinnamyl phenyl selenide **176a** with a copper (I) salt and bisoxazoline, the desired products were obtained with an enantioselectivity up to 34%. It was found that the introduction of an electron-withdrawing group (*o*-nitro or *o*-trifluoromethyl) into the phenylselenium moiety improved the enantioselectivity of the corresponding products, while the introduction of an electron-donating group (i.e., *o*-methoxy, ferrocenylselenium) inhibited the reaction completely. Analogously, the reactions of (*E*)-cinnamyl phenyl selenide **176a** with the Doyle catalyst [Rh_2_(5S-MEPY)_4_ (1 mol%)] were carried out. At 40 °C, the reaction proceeded smoothly with slightly better but still low enantioselectivity (up to 41% *ee*) in comparison to that obtained in the reaction with a copper catalyst (Scheme 46, Table 14).

**Scheme 47:**
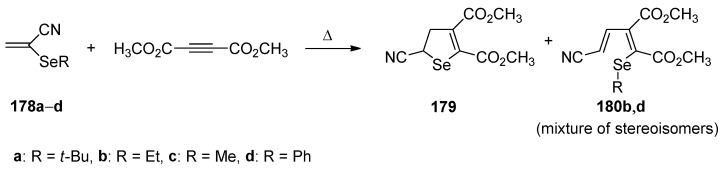
The reaction of 2-(organylseleno)propenenitriles **178a**–**d** with dimethyl ethynedicarboxylate.

The reaction of (2-*tert*-butylseleno)propenenitrile **178a** with an excess of dimethylethynedicarboxylate provided dimethyl 5-cyano-4,5-dihydroselenophene-2,3-dicarboxylate (**179**) in a 78% yield [80]. In turn, the reaction carried out with 2-(ethylseleno)-, 2-(methylseleno)- and 2-(phenylseleno)propenenitrile **178b**–**d** and dimethylethynedicarboxylate led to polysubstituted butadienes **180b**–**d** in 63%, 34%, and 46% yields, respectively (Scheme 47).

**Scheme 48:**
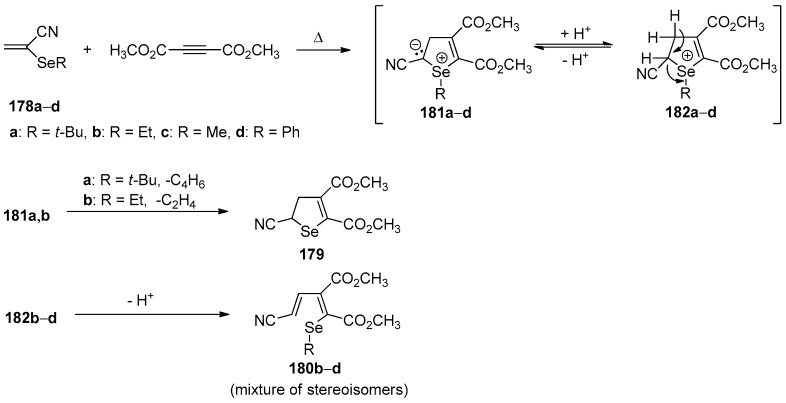
Reaction pathways for the formation of 2,3-dihydroselenophenes or polysubstituted butadienes from selenonium ylides.

Two different reaction pathways leading to compound **179** and/or **180b**–**d** were postulated. In both cases, the intermediate selenonium ylides **181a**–**d** formed in the reaction of **178a**–**d** and dimethylethynedicarboxylate are involved. When the R group on Se in substrate **178a**–**d** is an alkyl unit, alkene can be eliminated (isobutene from **178a**, ethene from **178b**). In the case of **178a**, elimination of isobutene appears very facile and compound **179** is formed as the only product. On the other hand, elimination of ethene from **178b** is retarded by ring opening to **180b**, via intermediate **182b**, which results in the formation of a mixture of products. In turn, for derivatives **178c**,**d,** no olefin can be eliminated and thus the ring opening occurred and led to **180c**,**d** (Scheme 48).

**Scheme 49:**
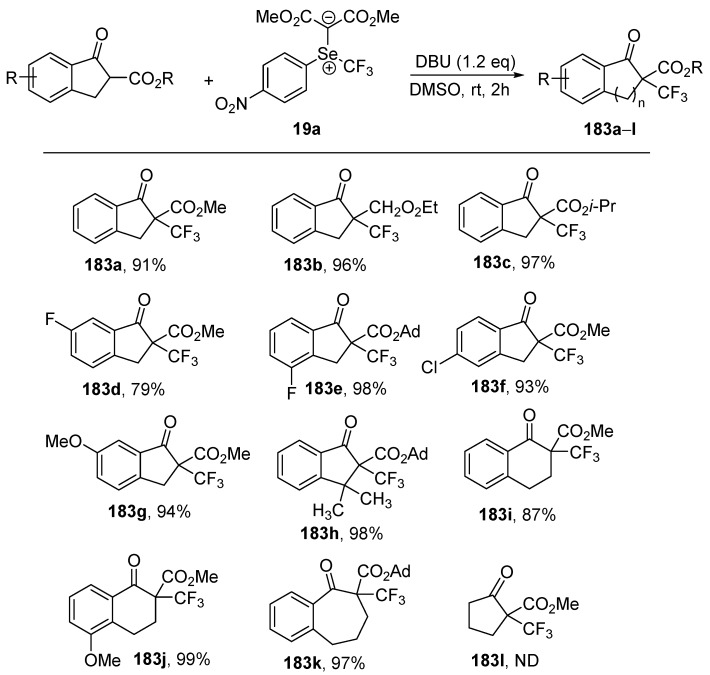
Reaction of β-ketoesters with **19a**.

Shen and coworkers described the application of trifluoromethyl-substituted selenonium ylide **19a** as an electrophilic reagent in the reactions with various nucleophiles, including β-ketoesters and silyl enol ethers, aryl/heteroaryl boronic acids, electron-rich heteroarenes, and sulfonates [38]. The reactions of various β-ketoesters derived from indanone, tetralone, or 1-benzosuberone with **19a** in the presence of DBU led to the corresponding trifluoromethylated compound **183a**–**k** in high yields (79–99%). The reactions of non-phenyl fused or open-chain β-ketoesters were much slower and the formation of the desired products were not observed (Scheme 49).

**Scheme 50:**
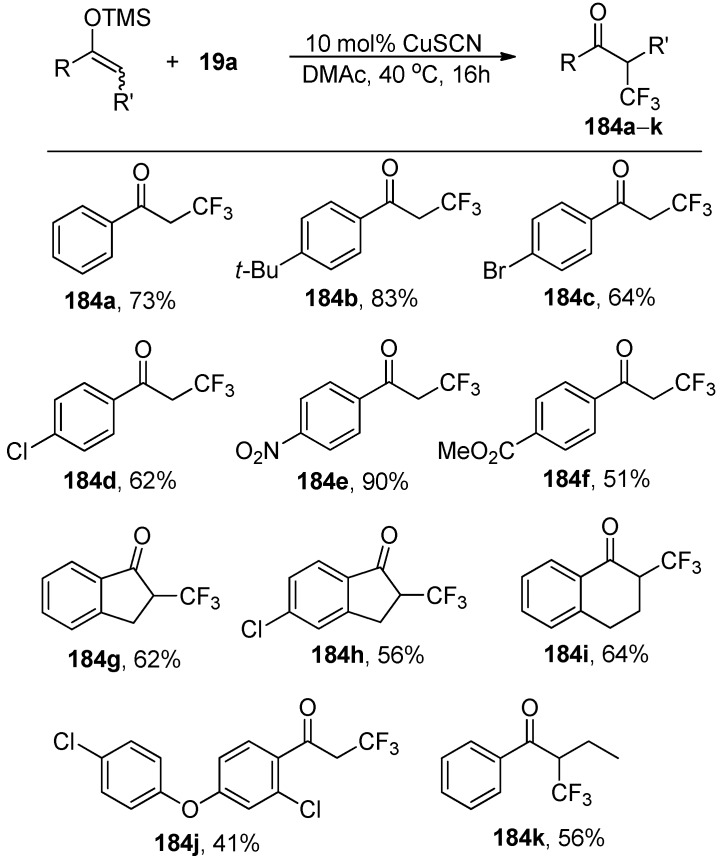
Reaction of silyl enol ethers with **19a**.

The reaction of trifluoromethyl-substituted selenium ylide **19a** with various silyl enolethers in the presence of 10 mol% CuSCN afforded the α-trifluoromethyl ketones **184a**–**k** in high yields (Scheme 50). The obtained results showed that **19a** is a good electrophilic trifluoromethylating reagent not only for β-ketoesters but also for silyl enol ethers.

**Scheme 51:**
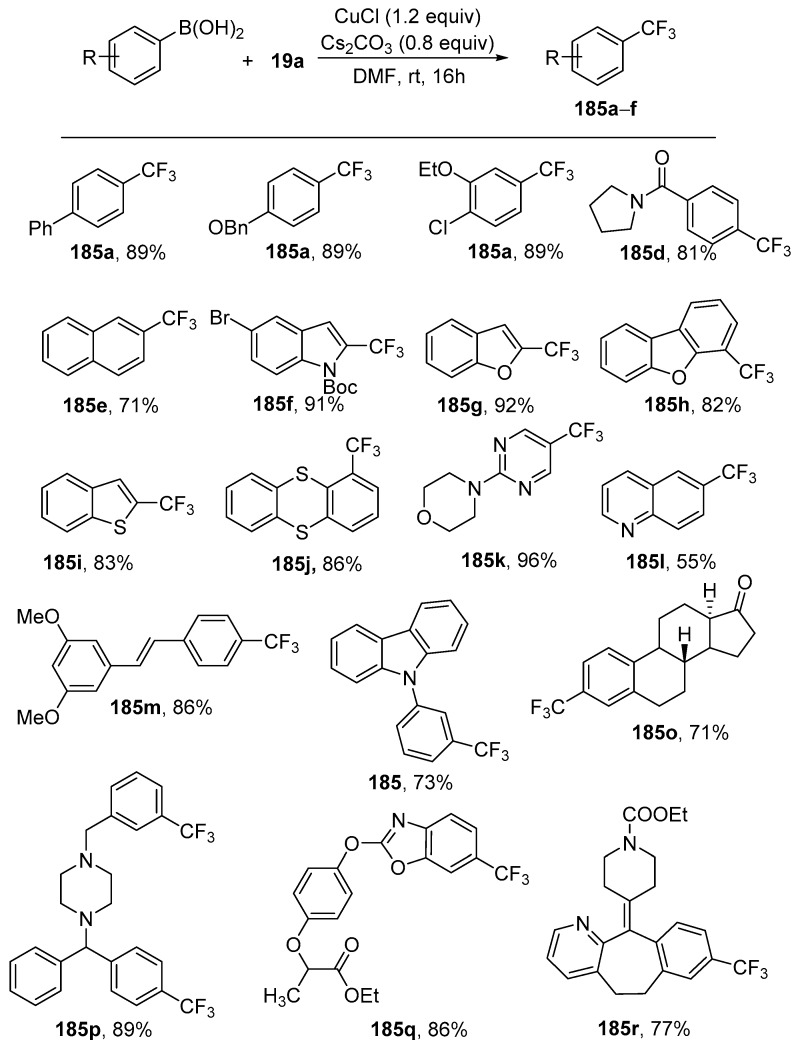
The copper-mediated reactions of aryl/heteroaryl boronic acids with **19a**.

Compound **19a** was also tested in the reaction with various arylboronic acids in the presence of a copper catalyst. The highest yields of **185a**–**r** were obtained when the reactions were carried out with the use of 1.2 equiv. of CuCl and 0.8 equiv. of Cs_2_CO_3_ in DMF at room temperature (Scheme 51). It is worth noting that trifluoromethylated heteroarenes are important structural units in many agrochemicals and they can be used in the preparation of drugs.

**Scheme 52:**
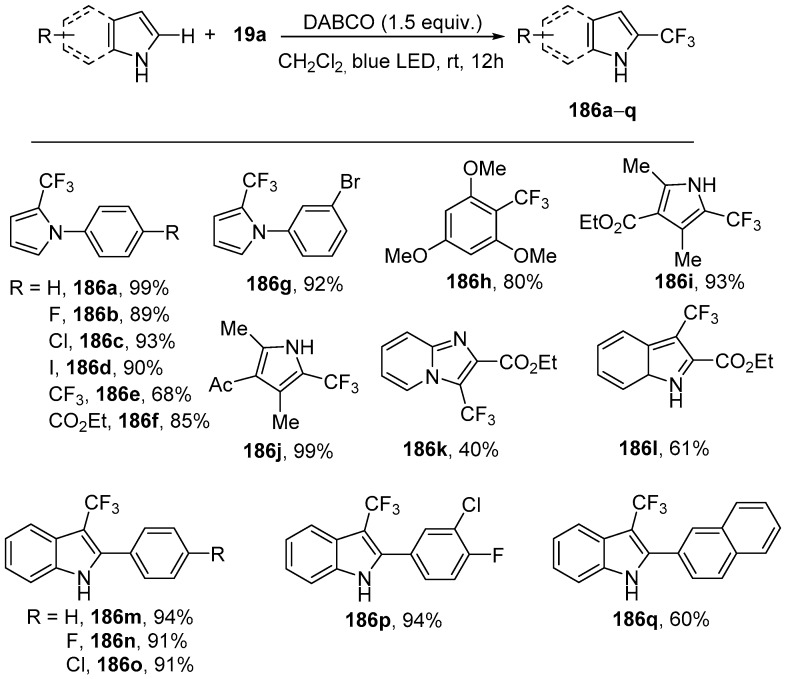
Visible light-promoted trifluoromethylation of the arene C–H bond with **19a**.

The application of reagent **19a** as a precursor of the trifluoromethyl radical was also studied. The obtained results showed that under the irradiation of blue LED light, reagent **19a** reacted with electron-rich indole or pyrrole derivatives in the presence of 1.5 equiv. of DABCO to give the *m*-trifluoromethylated indoles or *o*-trifluoromethylated pyrroles **186a**–**q** in high yields (Scheme 52). Similar reactions with the corresponding electron-rich arenes were less successful. It was found that the addition of DABCO played a key role in promoting the reaction since the absence of DABCO significantly decreased the yield to 40%.

**Scheme 53:**
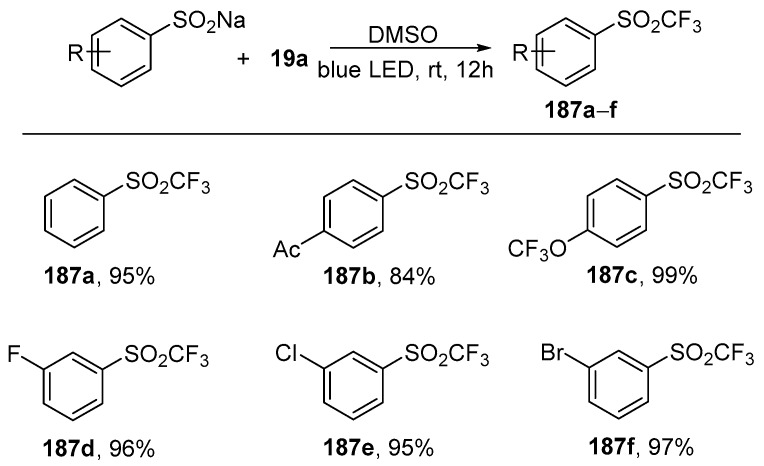
Visible light-promoted trifluoromethylation of sulfinates with **19a**.

Additionally, it was found that the trifluoromethyl radical could be generated in the presence of sodium benzenesulfinate instead of an amine. Under the irradiation with visible light of **19a**, the complex collapsed to generate the trifluoromethyl radical, which reacts with the sulfinate radical cation to form the trifluoromethylated sulfone derivatives. Under these conditions, various benzenesulfinate derivatives were trifluoromethylated to give trifluoromethylated sulfones **187a**–**f** in excellent yields (Scheme 53).

Another group discovered that the reaction of trifluoromethyl aryl selenonium ylides with aryl halides in the presence of copper salts provided diaryl selenides in good yields rather than the expected trifluoromethylated arenes [38].

**Scheme 54:**
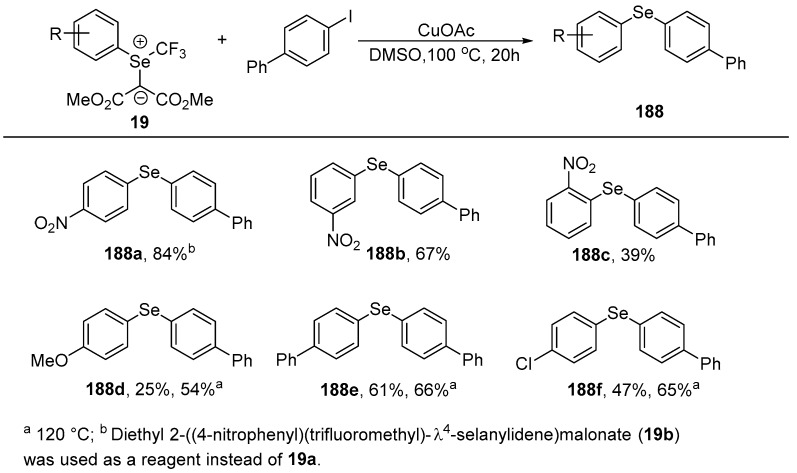
Cu-mediated arylselenylation of phenyl iodide by different trifluoromethyl aryl selenonium ylides **19a–i**.

Trifluoromethyl aryl selenonium ylides **19a**–**f** were suitable for the Cu-mediated arylselenylation of 4-iodo-1,1′-biphenyl (Scheme 54). A number of selenium ylides containing different substituents on the aromatic ring were tested. In most cases, the desired products were obtained in good to high yields (54–95%).

**Scheme 55:**
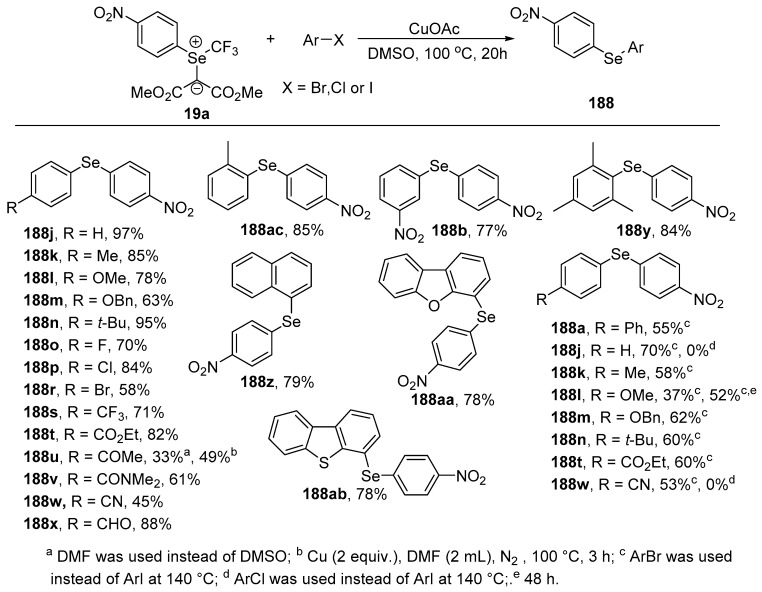
Cu-mediated arylselenylation of aryl halides by ylides **19**.

The reaction of dimethyl 2-((*p*-nitrophenyl)(trifluoromethyl)-λ^4^-selanylidene)malonate **19a** with various aryl iodides or bromides bearing electron-donating groups, electron-withdrawing groups on the aryl rings led to the corresponding arylselenylated products **188a**,**b** and **j-ac** in good to high yields (Scheme 55). When the reaction was carried out with aryl chlorides, such as chlorobenzene and *p*-chlorobenzonitrile, no desired products were obtained.

**Scheme 56:**
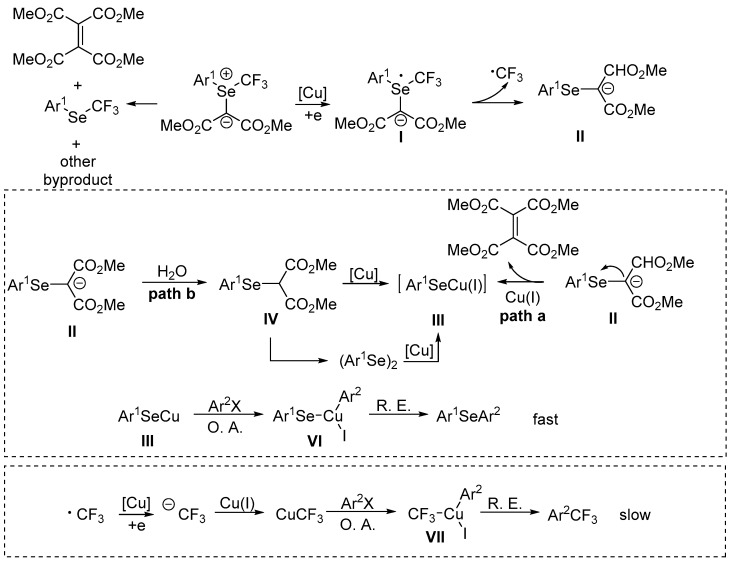
A plausible mechanism for Cu-mediated arylselenylation of aryl halides with trifluoromethyl aryl selenonium ylides **19**.

On the basis of the experiments and previous reports [81,82], a plausible reaction mechanism for the copper-mediated arylselenylation of aryl halides with trifluoromethyl aryl selenonium ylides was proposed (Scheme 56). In the first step, the reduction of trifluoromethyl aryl selenonium ylide by copper via single electron transfer takes place and a radical intermediate (**I**) is formed. Next, the intermediate (**I**) rapidly decomposes to release a ^•^CF_3_ radical and form an anion intermediate (**II**). Then, α-elimination of an intermediate (**II**) leads to an Ar^1^Se^−^ anion, which combines with Cu(I) salt to generate the Ar^1^SeCu complex (**III**) and release tetramethyl ethene-1,1,2,2-tetracarboxylate (path a). It is also possible that intermediate (**II**) undergoes protonation by traces of moisture in the solvent, which leads to dimethyl 2-(arylselanyl)malonate (**IV**) (path b). Reduction of compound **IV** by Cu afforded (phenylselanyl)copper (**III**) and/or diaryl diselenide (**V**). In the last step, Ar^1^SeCu complex (**III**) reacts with aryl halides via oxidative addition to give a Cu(III) complex (**VI**), which undergoes reductive elimination to form diaryl selenide and regenerate Cu(I) species. It is worth noting that the ^•^CF_3_ radical can be reduced via single electron transfer to the ^−^CF_3_ anion, which bonds to Cu(I) and forms CuCF_3_. Coupling of CuCF_3_ with aryl halides via oxidative addition and reductive elimination led to the trifluoromethylated product. Under standard reaction conditions, this pathway might be very slow and may not be the main process because only trace amounts of ArCF_3_ were detected.

**Scheme 57:**
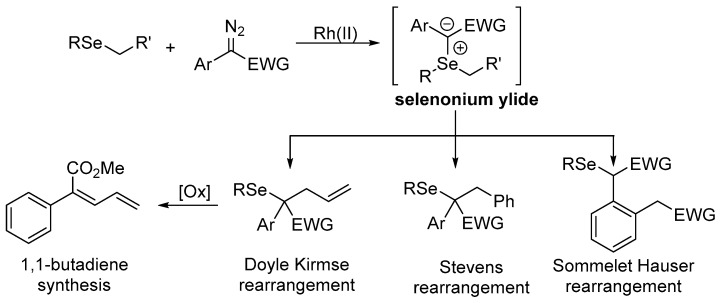
Rhodium-catalyzed generation and sigmatropic rearrangement of selenonium ylides and their synthetic applications.

Koengis and Jana recently reported the first study on rhodium-catalyzed generation and sigmatropic rearrangement of selenonium ylides and their synthetic applications. One of these applications allows access to important 1,1-disubstituted butadienes (Scheme 57) [83].

**Scheme 58:**
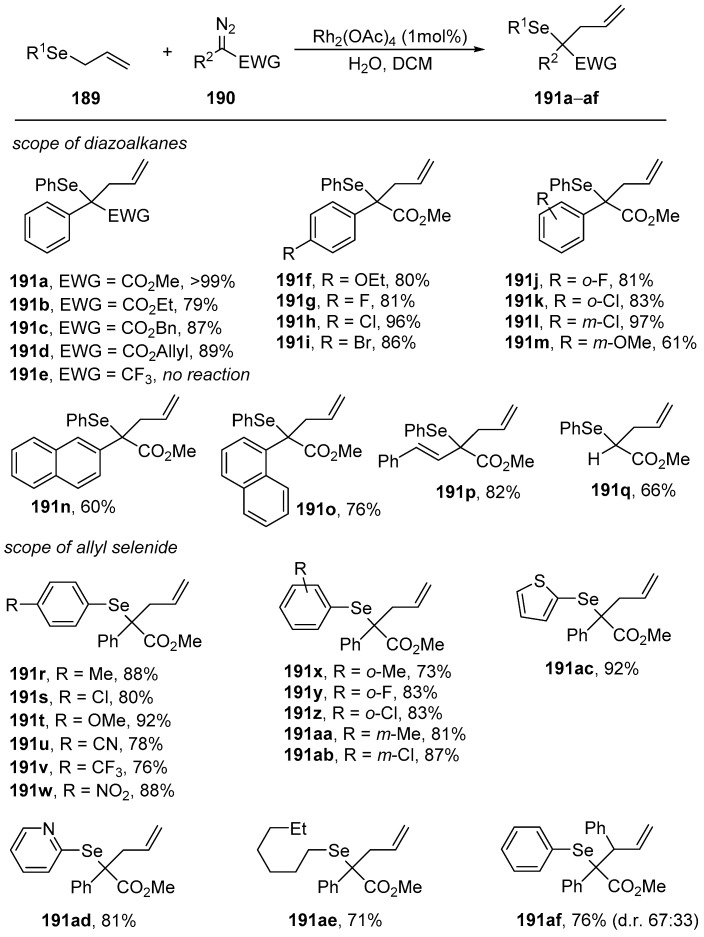
[2,3]-Sigmatropic rearrangement of selenium ylides generated in situ by a rhodium catalysed reaction of selenides **189** with diazocompounds **190**.

The most general application based on the generation of ylides in the reactions of allyl selenide **189** with different diazoesters **190** led to the desired homoallylic selenides **191** in excellent yields (Scheme 58). It was found that the type of substituent in the aromatic ring had little influence on the reaction yield, as various electron-donating groups as well as halides were compatible with the reaction. Additionally, other allylic selenides with a broad range of functional groups, such as nitro, cyano, and trifluoromethyl substituents, were investigated (Scheme 58). In this case, the seleno-Doyle–Kirmse reaction led to homoallylic selenides in good to high yields [83]. Thiophene and pyridine heterocycles were also compatible under these reaction conditions, and no poisoning of the rhodium catalyst occurred. The corresponding heterocycle-functionalized homoallylic selenides the rearrangement product in good yield but with only low diastereoselectivity, which is comparable to the diastereoselectivity range for the classic Doyle–Kirmse reactions [84,85,86].

**Scheme 59:**
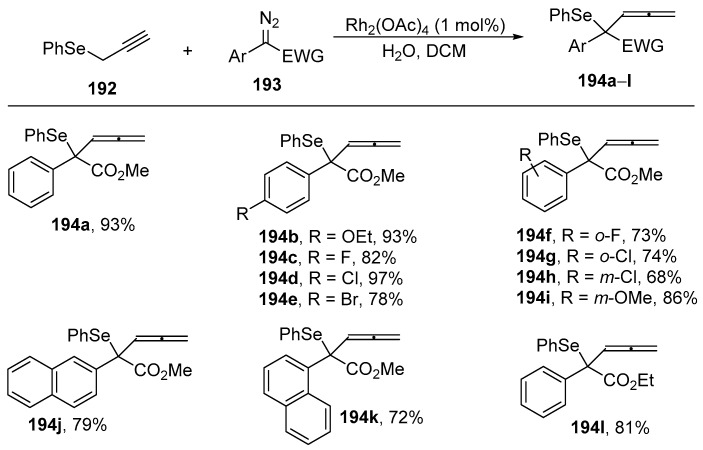
[2,3]-Sigmatropic rearrangement of selenonium ylides generated in situ by a rhodium-catalyzed reaction of selenides **192** with diazocompounds **193**.

Under optimized conditions, the reaction of propargyl selenides **192** with α-aryldiazoacetates **193** afforded the desired allenyl selenides **194a**–**l** in excellent yields (Scheme 59).

**Scheme 60:**
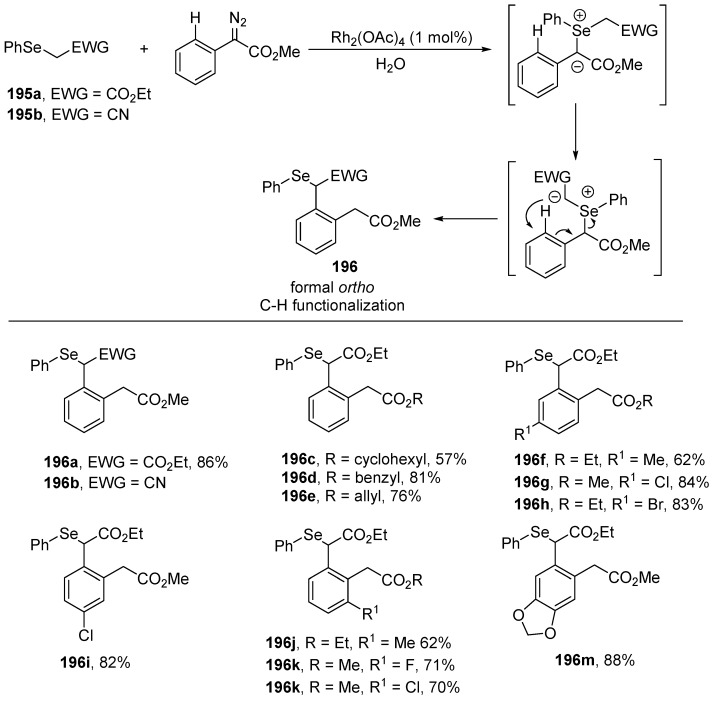
The Sommelet−Hauser rearrangement of selenonium ylides generated in situ by a rhodium-catalyzed reaction of selenides **195** with α-phenyldiazoacetate.

The use of ethyl 2-(phenylselanyl)acetate **195a** led to the α-seleno-substituted esters **196a**, as products of Sommelet−Hauser or [2,3]-sigmatropic rearrangement. The reaction product corresponds to a formal *o*-C-H functionalization of the aromatic substituent of **190**. This transformation also took place for the cyano-substituted selenide **195b** and afforded the products *o*-C-H functionalization in good to high yields. In turn, *meta*-substituted diazoalkanes reacted in a highly regioselective manner to form the corresponding trisubstituted phenyl acetic acid ester **196i** in high yield (Scheme 60).

**Scheme 61:**
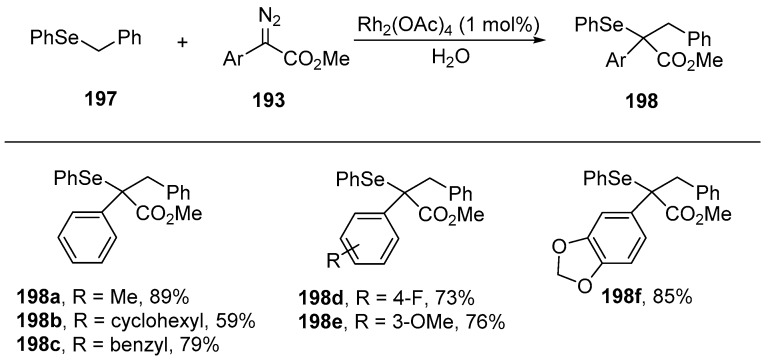
Rhodium-catalyzed reaction of benzyl phenyl selenide **197** with α-aryldiazoacetates **197**.

In the case of benzylic selenide **197**, the desired product of [1,2]-sigmatropic or Stevens rearrangement was obtained as a complex mixture. A change of the solvent used in the reaction to only water resulted in the rearrangement product **198a**–**f** in a good isolated yield. Similarly, the use of different donor−acceptor diazoesters smoothly led to the Stevens rearrangement product in a selective way (Scheme 61).

**Scheme 62:**
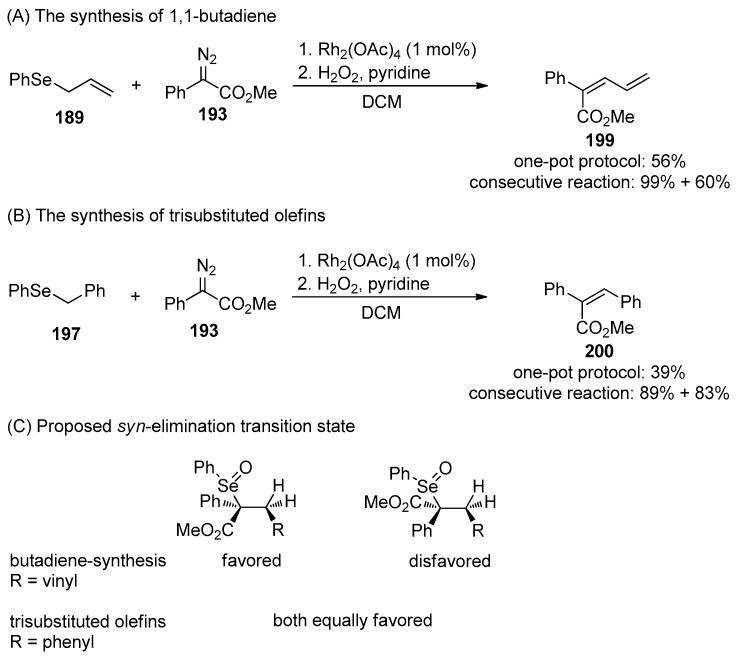
Applications of the in situ-generated selenonium ylides in the synthesis of olefins; (**A**) the synthesis of 1,1-butadiene; (**B**) the synthesis of trisubstituted olefins; (**C**) proposed *syn*-elimination state.

A one-pot protocol consisting of rhodium-catalyzed generation and rearrangement of selenonium ylides and a subsequent oxidation of the formed selenides in the synthesis of olefins is shown in Scheme 62. Thus, a two-step reaction of allyl selenide **189** with α-aryldiazoacetates **193** afforded *Z*-configured 1,1-disubstituted butadiene **199** in a 56% yield. A consecutive reaction protocol led to **199** in a similar yield (60%). The stereochemical outcome of this *syn* elimination was rationalized by the transition state in which the vinyl group and the phenyl ring have a *trans* conformation that results in the *Z*-configuration of the butadiene product (Scheme 62c). The stereochemistry of this reaction is complementary to that of the reaction of diazoalkanes with electrophilic palladium-allyl complexes [87]. A similar protocol was applied to the synthesis of trisubstituted olefin **200** as the product of the Stevens rearrangement of the generated ylide. The reaction led to the mixture of diastereoisomers in a 1:1 ratio (Scheme 62b), which is a result of the similar steric hindrance in both transition states (Scheme 62c).

**Scheme 63:**
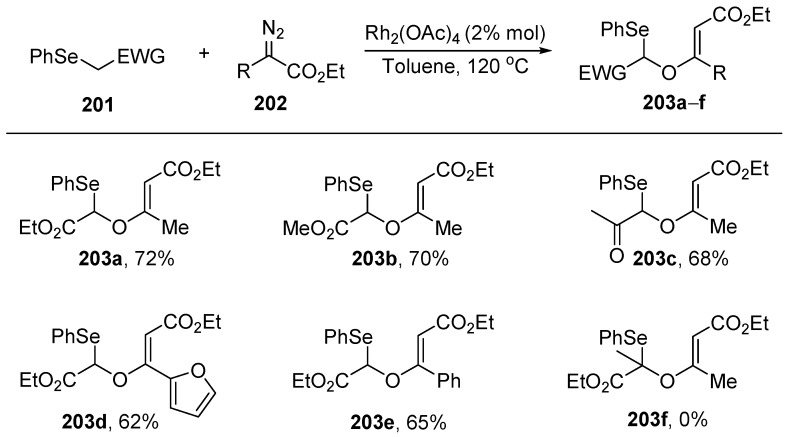
Rhodium-catalyzed arrangement of selenonium ylides.

In 2019, Anbarasan and coworkers described the rhodium-catalyzed rearrangement of selenonium ylides and their use in the synthesis of substituted vinylogous carbonates [88]. The reaction of various α-selenoesters **201** with diazocompound **202** in the presence of 2 %mol of Rh_2_(OAc)_4_ led to the desired products **203a**–**e** in a good yield (62–72%) (Scheme 63). Only in the case of ethyl α-phenylselenopropionate, the desired product **203f** was not observed, most probably due to the steric effect.

**Scheme 64:**
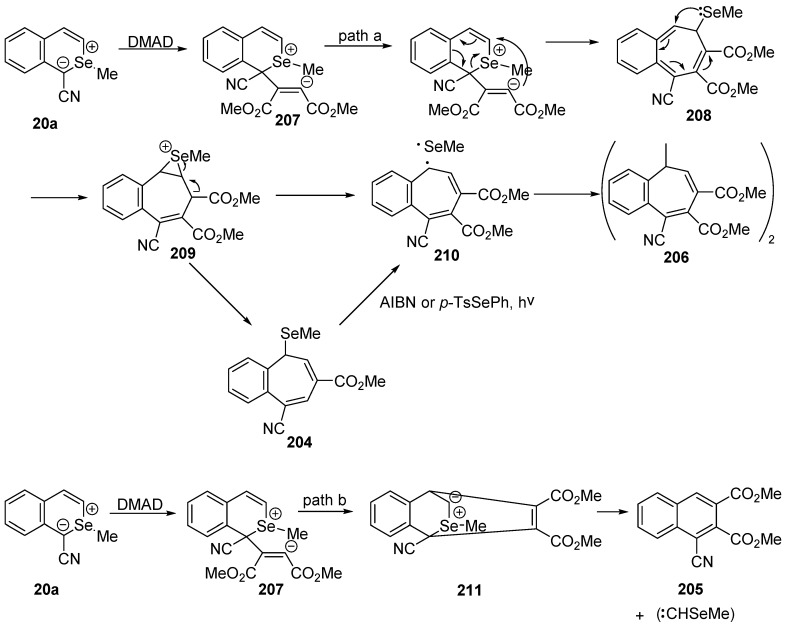
The plausible mechanism for the formation of benzocycloheptene **204** and naphthalene **205**.

Hori and coworkers reported the reactions of α-selenanaphthalene stabilized by the cyano group, i.e., l-cyano-2-methyl-2-selenanaphthalene (**20a**) [43]. Its reaction with dimethyl acetylenedicarboxylate (DMAD) in benzene at room temperature gave benzocycloheptene derivative **204** in a 61% yield, whereas the same reaction in sulfolane led to the mixture of naphthalene derivatives **205** (37%) and **204**. Furthermore, the reaction was carried out in acetonitrile produced **204** (17%) and bisbenzocycloheptenyl derivative **206** (56%). Scheme 64 shows a plausible mechanism for the formation of the benzocycloheptene **204** and the naphthalene **205**.

**Scheme 65:**
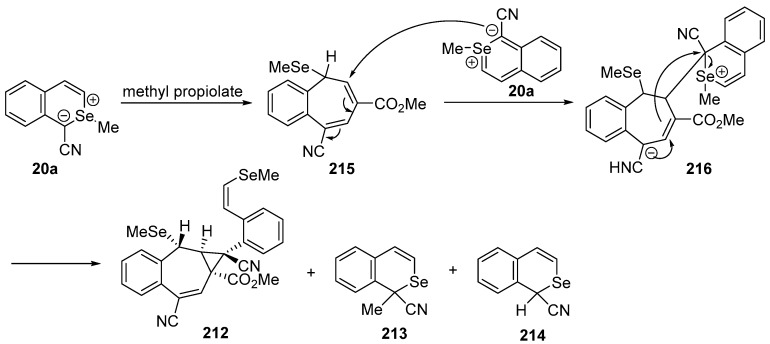
Reaction of selenanaphthalene **20a** with methyl propiolate.

In contrast to the reaction with DMAD, reaction of **20a** with methyl propiolate afforded a mixture of adduct **212**, rearranged product **213**, and demethylated product **214** (Scheme 65).

Moreover, the reactions of selenanaphthalene **20a** with olefins were examined (Table 15). The obtained results showed that the reaction of **20a** did not occur with styrene, dimethyl fumarate, and vinyl sulfones. However, when the reaction was conducted with vinyl sulfones, such as *trans*-styryl tolyl sulfone and 3-(*p*-tosyl)sulfolene, the yield of the 1,2-rearranged product **213** was reasonable.

**Scheme 66:**
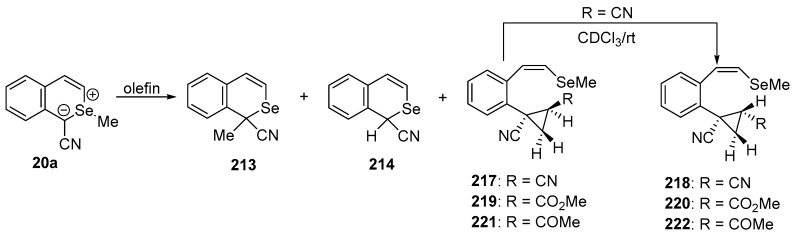
Reaction of selenanaphthalene **20a** with olefins.

Scheme 66 shows the reaction of selenanaphthalene **20a** with monosubstituted olefins. The reaction with acrylonitrile afforded *r*-l,*t*-2-dicyano-l-[2-(*cis*-2-methylselenovinyl)-phenyl] cyclopropane (**217**) and the *r*-l,*c*-2-dicyano isomer (**218**) in 29% and 53% yields, respectively. Methyl acrylate and methyl vinyl ketone reacted similarly with **7** to afford *cis*-*trans* mixtures of the cyclopropane derivatives **219**, **220**, **221**, and **222**, respectively.

## 5. Conclusions and Perspectives

Synthetic approaches to the preparation of achiral and chiral selenonium ylides (including a few optically active ones) and the problem of their structural features, synthesis, reactivity, and application in organic synthesis were described in this manuscript. The purpose of this mini review was to provide the available information on all topics mentioned above in order to stimulate additional research in this field. The rationale for this research topic is the structural similarity between selenonium ylides and sulfonium ylides, which play a very important role as new synthetic reagents, biologically active compounds, and new functional materials [4,5,6,7,8]. Therefore, it is reasonable to expect that achiral and optically active selenonium ylides should be just as useful as sufonium ylides when they have sufficiently high chemical and optical stability. It is reasonable to expect that further research will allow the preparation of model compounds containing, for example, more sterically demanding substituents, which in turn enable the preparation of optically active selenonium ylides with an optical stability comparable to sulfonium ylides. First of all, it is worth checking their preparation by asymmetric synthesis. Moreover, access to a wider group of selenonium ylides, achiral and optically active, should open the way to the isolation of the corresponding oxoselenonium ylides, which, to our best knowledge, have not been reported in the chemical literature so far.

## Figures and Tables

**Table 1:** Synthesis of *cis*-(1*R*,2*R*,3*R*)-trisubstituted cyclopropanes **78** from *exo*-camphor-derived selenonium ylides **75a**,**b**. ^a^

Entry	Ylide 75	77	Product, (Yield, %) ^b^	*cis*/*trans*^c^	*ee* [%] ^d^
1	**75a**	**77a**	**78a**, (92)	>99:1	91
2	**75a**	**77b**	**78b**, (91)	>99:1	>99
3	**75a**	**77c**	**78c**, (87)	98:2	92
4	**75a**	**77d**	**78d**, (70)	90:10	>99
5	**75a**	**77e**	**78e**, (86)	>99:1	95
6	**75a**	**77f**	**78f**, (93)	>99:1	93
7	**75a**	**77g**	**78g**, (86)	90:10	>99
8	**75a**	**77h**	**78h**, (83)	95:5	93
9	**75a**	**77i**	**78i**, (90)	97:3	91
10	**75a**	**77j**	**78j**, (85)	92:8	92
11	**75a**	**77k**	**78k**, (92)	>99:1	>99
12	**75a**	**77l**	**78l**, (81)	95:5	95
13	**75b**	**77a**	**78m**, (90)	98:2	87
14	**75b**	**77c**	**78n**, (84)	97:3	81
15	**75b**	**77f**	**78o**, (91)	>99:1	90
16	**75b**	**77i**	**78p**, (80)	90:10	81

^a^ The reaction was performed with the ratio of salt/base/**75** 1:2:1 at −78 °C for 3–5 h. ^b^ Isolated yields. ^c^ Determined by ^1^H-NMR or GC. ^d^ Determined by chiral HPLC using Chiracel OD-H or AD-H column.

**Table 2:** Synthesis of *cis*-(1*S*,2*S*,3*S*)-trisubstituted cyclopropanes **78′** from *endo*-camphor selenonium ylide **76**. ^a^

Entry	77	Product (Yield, %) ^b^	*cis*/*trans*^c^	*ee* [%] ^d^
1	**77a**	**78′a**, (90)	95:5	>99
2	**77b**	**78′b**, (88)	>99:1	95
3	**77c**	**78′c**, (83)	98:2	>99
4	**77e**	**78′e**, (84)	>99:1	85
5	**77g**	**78′g**, (82)	90:10	97
6	**77h**	**78′h**, (81)	98:2	70
7	**77i**	**78′i**, (90)	97:3	94
8	**77l**	**78′l**, (81)	95:5	>99

^a^ The reaction was performed with the ratio of salt/base/**76** 1:2:1 at −78 °C for 3–5 h. ^b^ Isolated yields. ^c^ Determined by ^1^H-NMR or GC. ^d^ Determined by chiral HPLC using Chiracel OD-H or AD-H column.

**Table 3:** Stereoselective synthesis of *trans*-(1*R*,2*R*,3*S*)-trisubstituted cyclopropanes **80**. ^a^

Entry	81	Product, (Yield, %) ^b^	*cis*/*trans*^c^	*ee* [%] ^d^
1	**81a**	**80a**, (93)	98:2	81
2	**81b**	**80b**, (92)	97:3	97
3	**81c**	**80c**, (89)	98:2	99
4	**81d**	**80d**, (91)	>99:1	98
5	**81e**	**80e**, (90)	95:5	98
6	**81f**	**80f**, (80)	>99:1	51
7	**81g**	**80g**, (82)	96:4	85
8	**81h**	**80h**, (83)	96:4	81

^a^ The reaction was performed with the ratio of salt/base/**79** 2.5:3:1 at −78 °C for 4–5h. ^b^ Isolated yields. ^c^ Determined by ^1^H-NMR or GC. ^d^ Determined by chiral HPLC using Chiracel OD-H or AD-H column.

**Table 4:** Cyclopropanation reaction with selenonium salt containing a benzyl terpenyl substituent.

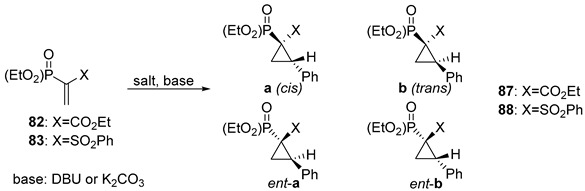						
Entry	Salt	Michael Acceptor	Reaction Condition	*trans*/*cis*	Ratio of *trans* Diastereomers l/h	Ratio of *cis* Diastereomers l/h
1	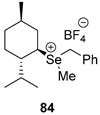	**82**	K_2_CO_3_, CH_2_Cl_2_	47.0:53.0 ^a^	43.5/56.5	40.3/59.7
2	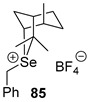	**82**	DBU, acetone	56.0:44.0 ^a^	5.9/94.1	9.4/90.6
3	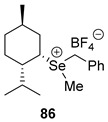	**83**	K_2_CO_3_, CH_2_Cl_2_	80.0:20.0 ^a^	— ^b^	— ^b^
4	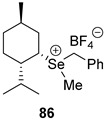	**83**	DBU, MeCN	76.2:23.8 ^a^	63.3/36.7	78.5/21.5

l—Lower R_f_ in HPLC; h—Higher R_f_ in HPLC. ^a^ The ratio of diastereomers determined by NMR. ^b^ Inseparable impurities disturbed the precise determination of the enantiomeric ratio.

**Table 5:** Cyclopropanation reaction with selenonium salt containing the ethyl acetate substituent.

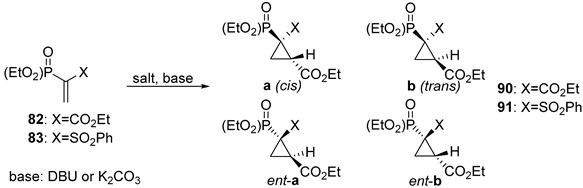						
Entry	Salt	Michael Acceptor	Reaction Condition	*trans*/*cis*	Ratio of *cis* Diasereomers l/h	Ratio of *trans* Diasereomers l/h
1	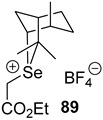	**82**	DBU, MeCN	65.0:35.0 ^a^	98.0/2.0 ^a^	99.0/1.0
2	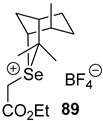	**83**	DBU, MeCN	72.5:27.5 ^a^	1.6/98.4	2.5/97.5

l—Lower R_f_ in HPLC; h—Higher R_f_ in HPLC. ^a^ The ratio of diastereomers determined by NMR.

**Table 6:** Reaction of selenonium salts **92a**–**c** with various aldehydes and ketones.

Entry	Selenonium Salt	Aldehyde or Ketone		Epoxides 94–102 (Yield, %)
		R^3^	R^4^	
1	**92a**	C_6_H_5_	H	**94**, (90)
2	**92a**	*p*-CH_3_-OC_6_H_14_	H	**95**, (75)
3	**92a**	*p*-CN-C_6_H_14_	H	**96**, (54)
4	**92a**	mesityl	H	**97**, (79)
5	**92a**	C_6_H_5_CH=CH	H	**98**, (71)
6	**92a**	C_6_H_5_CH=CH	C_6_H_5_	**99**, (50)
7	**92b**	C_6_H_5_	H	**100**, (90)
8	**92a**	C_6_H_5_	C_6_H_5_	**101**, (90)
9	**92c**	C_6_H_5_	H	**102**, (69)

**Table 7:** Electrochemical reduction of substituted methyltetrahydroselenonium bromide **103a**–**g** in the presence of benzaldehyde.

Entry	Selenonium Salt	Reaction	Product (Yield, %)
1	**103a**	Epoxidation	**104** (79)
2	**103b**	Epoxidation	**105** (63)
3	**103c**	Radical coupling	**109** (68)
4	**103d**	Radical coupling	**110** (72)
5	**103e**	Epoxidation	**106** (69)
6	**103f**	Epoxidation	**107** (30)
7	**103g**	Epoxidation	**108** (32)

**Table 8:** Electrochemical reduction of selenonium salts **103a**–**g** without benzaldehyde.

Entry	Selenonium Salt	Reaction	Product (Yield, %)
1	**103a**	Sigmatropic rearrangement	**111** (31)
2	**103b**	Sigmatropic rearrangement	**112** (38)
3	**103c**	Radical coupling	**109** (73)
4	**103d**	Radical coupling	**110** (94)
5	**103e**	Sigmatropic rearrangement	**113** (50)
6	**103f**	Sigmatropic rearrangement	**114** (70)
7	**103g**	Carbene formation	**115** (36), **116** (21)

**Table 9:** Reaction time and yields of oxiranylketones **135a**–**j** obtained in the reaction of ketoselenonium ylides **134a**–**c** with aromatic aldehydes.

Entry	134	Aldehyde R^1^CHO	Time (h)	Product (Yield, %)
1	**134a**	R^1^ = *p*-ClC_6_H_4_	12	**135a** (78)
2	**134a**	R^1^ = *p*-NO_2_C_6_H_4_	3.5	**135b** (84)
3	**134a**	R^1^ = *o*-NO_2_C_6_H_4_	1.5	**135c** (71)
4	**134a**	R^1^ = *m*-NO_2_C_6_H_4_	2	**135d** (58)
5	**134a**	R^1^ = *p*-BrC_6_H_4_	9	**135e** (55)
6	**134a**	R^1^ = *o*-BrC_6_H_4_	2	**135f** (92)
7	**134a**	R^1^ = *m*-BrC_6_H_4_	5	**135g** (90)
8	**134a**	R^1^ = Ph	6.5	**135h** (40)
9	**134b**	R^1^ = *p*-NO_2_C_6_H_4_	3	**135i** (89)
10	**134c**	R^1^ = *p*-NO_2_C_6_H_4_	3	**135j** (71)

**Table 10:** Reaction time and yields of oxiranylketones **136a**–**e** obtained from ketoselenonium ylide **134a** and aliphatic aldehydes.

Entry	Aldehyde R^2^CHO	Time	Product (Yield, %)
1	R^2^ = CH_3_CH_2_	overnight	**137a** (18)
2	R^2^ = CH_3_(CH_2_)_2_	24 h	**137b** (34)
3	R^2^ = Ph(CH_2_)_2_	overnight	**137c** (54)
4	R^2^ = (CH_3_)_2_CH	overnight	**137d** (37)
5	R^2^ = (CH_3_)CHCH_2_	24 h	**137e** (63)

**Table 11:** Aziridines **137a**–**d** obtained in the reaction of alkynylselenonium salt **134a** with aromatic aldehydes and TsNHNa.

Entry	Aldehyde	Solvent	Product (Yield, %)
1	R^1^ = *p*-ClC_6_H_4_	CH_2_Cl_2_-MeCN	**137a** (44)
2	R^1^ = *p*-NO_2_C_6_H_4_	MeCN	**137b** (44)
3	R^1^ = *o*-BrC_6_H_4_	MeCN	**137c** (48)
4	R^1^ = *o*-MeOC_6_H_4_	CH_2_Cl_2_-MeCN	**137d** (30)

**Table 12:** Reactions of vinylselenonium salt **140a** with aldehydes in the presence of KH. ^a^.

Entry	RCHO	Reaction Conditions	Product (yield, %)
1	*p*-NO_2_C_6_H_4_CHO	KH, THF, 0 °C, 0.5 h	**141a** (94)
2	*p*-ClC_6_H_4_CHO	KH, THF, 0 °C, 0.5 h	**141b** (77)
3	*p*-BrC_6_H_4_CHO	KH, THF, 0 °C, 0.5 h	**141e** (76)
4	PhCHO	KH, THF, −10 °C, overnight	**141f** (43)
5	PhCHO	KH, THF-DMSO,^b^ −30 °C, 2 h	**141f** (60)
6	*p*-MeC_6_H_4_CHO	KH, THF-DMSO,^b^ −30 °C, 3.5 h	**141g** (49)

^a^**141a**: RCHO : KH = 2 : 1.3. ^b^ THF : DMSO = 13 : 1.

**Table 13:** Reaction conditions and the yields of alkylidenecyclopropanes **164a**–**c**.

Entry	Method	Product 164 (Yield, %)
**162a**	A then D	**164a** (85)
**162a**	C (MeI) then D	**164a** (53)
**162b**	C (MeI) then D	**164b** (60)
**162b**	C (MeI) then D	**164b** (56)
**162c**	B then D	**164c** (67)
**162c**	A then D	**164c** (85)

**Table 14:** Reaction conditions for Scheme 46: Cu: CuOTf (5 mol%) + bisoxazoline (5 mol%); ii, cat. Rh: Rh_2_(5*S*-MEPY)_4_ (1 mol%). Tf = SO_2_CF_3_.

Entry	176	Reaction Conditions ^a^	Yield (%)	dr of 177 ^b^ (*ee*, %)
1	**176a** (Ar = C_6_H_5_)	Cu, CHCl_3_, 0 °C, 24 h	71	66:34 (22, 32)
2	**176b** (Ar = *o*-NO_2_C_6_H_4_)	Cu, CHCl_3_, 0 °C, 24 h	55	53:47 (13, 34)
3	**176c** (Ar = *o*-CCF_3_C_6_H_4_)	Cu, CHCl_3_, 0 °C, 24 h	35	69:31 (8, 30)
4	**176a**	Rh, CH_2_Cl_2_, 40 °C, 20 h	65	58:42 (25, 41)
5	**176a**	Rh, CH_2_Cl_2_, 15 °C, 20 h	10	-

^a^ Cu: CuOTf (5 mol%) + bisoxazoline (5 mol%); Rh: Rh_2_(5*S*-MEPY)_4_ (1 mol%); ^b^ Determined by ^l^H-NMR.

**Table 15:** Reactions of selenanaphthalene **20a** with electron-deficient olefins.

Entry	Olefins (equiv.)	Solvent	Products (yield, %)
1	(*E*)-styryl *p*-tolyl sulfone (1.7)	MeCN	**213** (61)
2	(*E*)-styryl *p*-tolyl sulfone (1.7)	C_6_H_6_	**213** (59)
3	(*E*)-styryl *p*-tolyl sulfone (0.1)	C_6_H_6_	**213** (18), **214** (14)
4	3-(*p*-tosyl)sulfone (1.7)	MeCN	**213** (66), **214** (6)
5	styrene (1.7)	C_6_H_6_	**213** (17), **214** (9),
6	acrylonitrile (1.1)	MeCN	**214** (trace), **217** (29), **218** (53)
7	methyl acrylate (1.1)	MeCN	**213** (13), **219** (18), **220** (33)
8	methyl vinyl ketone (1.1)	MeCN	**213** (3), **221** (37), **222** (55)
